# Al-Ansab and the Dead Sea: Mid-MIS 3 archaeology and environment of the early Ahmarian population of the Levantine corridor

**DOI:** 10.1371/journal.pone.0239968

**Published:** 2020-10-13

**Authors:** Jürgen Richter, Thomas Litt, Frank Lehmkuhl, Andreas Hense, Thomas C. Hauck, Dirk F. Leder, Andrea Miebach, Hannah Parow-Souchon, Florian Sauer, Jonathan Schoenenberg, Maysoon Al-Nahar, Shumon T. Hussain

**Affiliations:** 1 Institute of Prehistoric Archaeology, University of Cologne, Cologne, Germany; 2 Institute of Geosciences, Palaeontology Section, University of Bonn, Bonn, Germany; 3 Department of Geography, RWTH Aachen University, Aachen, Germany; 4 Institute for Geosciences, Meteorology Section, University of Bonn, Bonn, Germany; 5 Department of Archaeology, Lower Saxony State Office for Cultural Heritage, Hanover, Germany; 6 Department of Archaeology, University of Jordan, Amman, Jordan; 7 Department of Archaeology and Heritage Studies, School of Culture and Society and BIOCHANGE - Center for Biodiversity Dynamics in a Changing World, Institute for Bioscience, Aarhus University, Aarhus, Denmark; Universita degli Studi di Milano, ITALY

## Abstract

Our field data from the Upper Palaeolithic site of Al-Ansab 1 (Jordan) and from a pollen sequence in the Dead Sea elucidate the role that changing Steppe landscapes played in facilitating anatomically modern human populations to enter a major expansion and consolidation phase, known as the „Early Ahmarian“, several millennia subsequent to their initial Marine Isotope Stage 4/3 migration from Africa, into the Middle East. The Early Ahmarian techno-cultural unit covers a time range between 45 ka–37 ka BP. With so far more than 50 sites found, the Early Ahmarian is the first fully Upper Palaeolithic techno-cultural unit exclusively and undisputedly related to anatomically modern human populations. In order to better understand the potentially attractive features of the Early Ahmarian environmental context that supported its persistence for over 8,000 years, we carried out a decennial research program in Jordan and in the Dead Sea. This included (1) a geoscientific and archaeological survey program in the Wadi Sabra (Jordan) with a particular focus on excavations at the Early Ahmarian site of Al-Ansab 1 alongside the detailed analysis of Quaternary sediments from the same area and (2) palaeobotanical research based on Quaternary lake deposits from the Dead Sea. Our pollen data from the Dead Sea indicate slow, low frequency vegetational variation with expanding *Artemisia* steppe, from 60 to 20 ka BP (MIS 3–2). Here, we see a reciprocal assimilation of southern and northern Levantine vegetation zones thereby enhancing a long-lasting south-to-north steppe corridor. The same integration process accelerated about 40 ka ago, when forested areas retreated in the Lebanese Mountains. The process then extended to encompass an area from Southern Lebanon to the Sinai Peninsula. We argue that, at the same time, the carriers of the Early Ahmarian techno-cultural unit extended their habitat from their original Mediterranean biome (in the North) to the Saharo-Arabian biome (to the South). Our excavation of Al-Ansab 1, a campsite at the eastern margins of the Early Ahmarian settlement area, indicates far reaching annual movements of small, highly mobile hunter-gatherer groups. We assume a low degree of settlement complexity, still allowing for habitat extension of the Early Ahmarian into the margins of the Levantine corridor. Due to our radiometric dates, our combined archaeological and environmental record sheds light on an evolved phase of the Early Ahmarian, around 38 ka ago, rather than the starting phase of this techno-cultural unit. Possible application of our model to the starting phase of the Early Ahmarian remains an aspect of future research.

## Introduction

**Fig 1:**
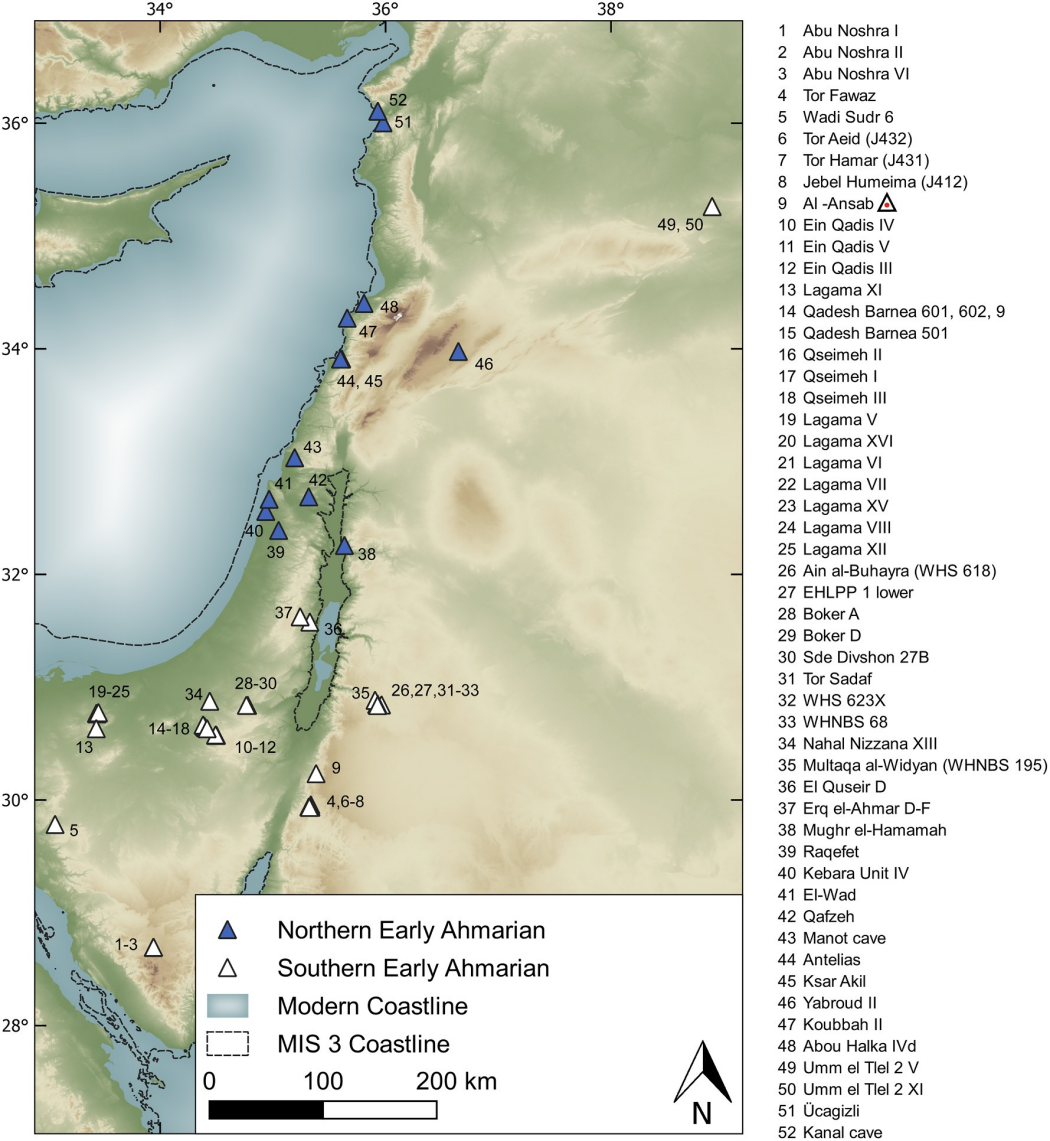
Archaeological sites attributed to the Early Ahmarian techno-cultural unit composed of the Northern Early Ahmarian (NEA) and Southern Early Ahmarian (SEA) groups. SEA sites include the Lagaman regional variant [4] known in the Negev Desert and on the Sinai Peninsula (see S1 Table for detailed site catalogue, coordinates and references). Coordinate system used: WGS 84/EPSG 4326.

During Marine Isotope Stage (MIS) 3—particularly between 45 ka cal BP and 37 ka cal BP—the Early Ahmarian techno-cultural unit covered the areas today known as the Sinai Peninsula, the Negev Desert, the Wadi Araba, western Transjordan, the Lebanon Mountains and the southern Syrian desert (Fig 1). To date, we know more than 50 sites in this area (S1 Table). The Abu Noshra sites (Sinai) constitute the southernmost and Üçağızlı Cave and Kanal Cave (Turkey) the northernmost Ahmarian occurrences [1]. The Ahmarian was defined more than 30 years ago as an Early Upper Palaeolithic blade industry characterized by the presence of so-called “El-Wad points” (pointed bladelets with marginal retouch) and some other typo-technological features in the lithic assemblages [2, 3]. Setting aside the controversial debate about the possibly longer duration of the Ahmarian techno-cultural unit [4], in this paper we focus on the early occurrences beginning c. 45 ka cal BP and the following 8,000 years, commonly named the “Early Ahmarian” [4].

The Early Ahmarian has been subdivided into a northern and a southern variant, the Northern Early Ahmarian (NEA) and Southern Early Ahmarian (SEA) (Fig 1). Given the supposed similarities between the Early Ahmarian and the European Protoaurignacian [5], the Early Ahmarian appears to be part of the crucial phase when the Upper Palaeolithic began all over Western Eurasia and Anatomically Modern Humans (AMH) expanded northwards towards Europe and beyond [cf. 4]. During the last decade, the Early Ahmarian has attracted particular attention given its role as the first techno-cultural unit in the Near East clearly related to AMH and not Neanderthals [6–8]. Its chronological position, after the Neanderthal/AMH chronological boundary (around 55 ka BP [9–11]), and its onset concurrent with the earliest AMH occurrence in Europe (45–43 ka BP [12]) rise questions regarding the adaptational system connected with the Early Ahmarian [13, 14]. Both the anthropological and cultural puzzle of the initial dispersal of AMH in the Middle East and the subsequent Early Ahmarian technocultural phenomenon, exclusively connected with AMH have provoked extensive research into the environmental and climatic context at mid-MIS 3 [cf. 15–17].

### Research frame

Our own theoretical framework of the „contextual area“-approach [18] is designed to unravel human-environment relationships, along the geographic trajectory of AMH migration from Africa to the Levant, on three different scales: (a) occupational sites of AMH and their nearby surroundings (local scale), the goal being to pinpoint the context of Early Ahmarian adaptation within a local, long-term environmental sequence (MIS 3); (b) the territory (regional scale), to reconstruct an annual mobility range of the bearers of the Early Ahmarian techno-cultural unit; (c) the contextual area (supraregional scale), to place the general area of Early Ahmarian occupation into related biomes.

Since 2009, the Jordan Rift Valley, with fieldwork localities in the Wadi Sabra/Jordan and in the Dead Sea, was chosen for intensive field research, under the umbrella of the Collaborative Research Centre (CRC) 806 „Our Way to Europe”[19]. The centre aims to elucidate the routes of early AMH from Africa into Europe by producing and bringing together environmental data (mostly from freshwater lake sediments and terrestrial archives) and archaeological data. This is achieved according to the mentioned concept of “contextual areas” [18, 20] in order to identify areas or regions of systemic coherence, at a given time slice and within natural boundaries relevant to human adaptation.

### Research questions

The 55–43 ka BP time range representing the first manifestation of AMH in the Middle East [9, 11, 12], the subsequent Early Ahmarian illustrates eight millennia of further expansion and successful consolidation of AMH in the area between 45 ka– 37 ka BP. Consequently, the set of behavioral and technological properties unique to the Early Ahmarian techno-cultural unit must have played a role in the sustainable adaptation and consolidation of AMH populations to post-45 ka BP Middle East environments. Therefore, we focus on three main research questions: (1) Which adaptational strategies of the bearers of the Early Ahmarian techno-cultural unit facilitated the further establishment and dispersal of AMH in the region, (2) which environmental conditions prevailed during the eight millennia of the Early Ahmarian, 45–37 ka BP and (3) which regional trends are tentatively visible in the cultural and environmental evolution of the time?

As environmental and archaeological archives needed were not to be found a single location, we carried out fieldwork at two research locales: archaeological and geological fieldwork focused on the Lower Wadi Sabra and palaeobotanical field data were obtained in the Dead Sea.

### First research locale: Lower Wadi Sabra

**Fig 2:**
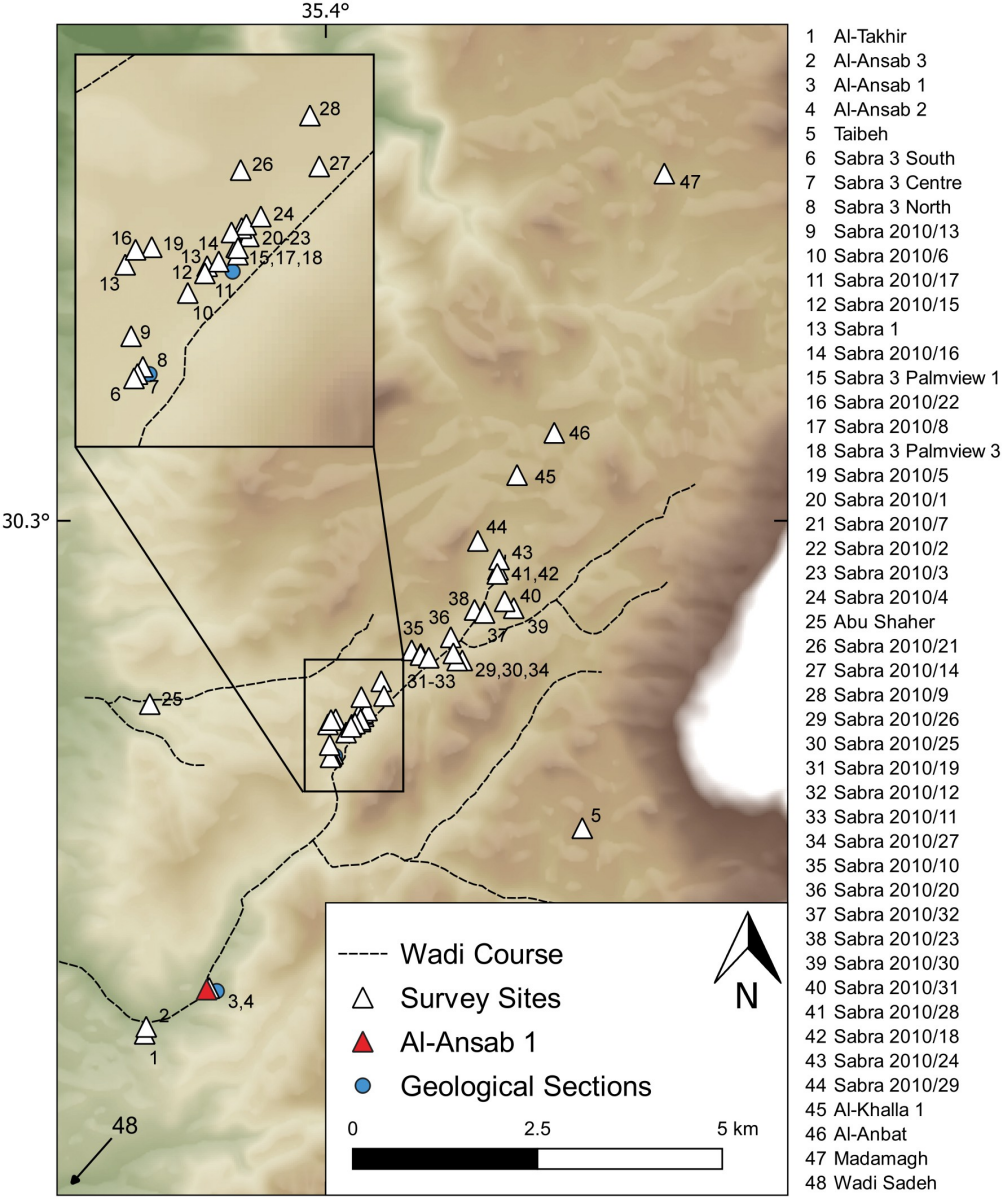
Palaeolithic sites discovered in Wadi Sabra during the CRC 806 archaeological survey from 2009 to 2019. Coordinate system used: WGS 84/EPSG 4326.

The Al-Ansab 1 archaeological site, in the Lower Wadi Sabra, around 15 km south of Petra (Jordan), is among the few known stratified Early Ahmarian open air settlements available to modern excavation. Forming a window into the mid-MIS 3 local environmental sequence of the Lower Wadi Sabra (Fig 2, site numbers 1–4), the 20 m of valley fill underlying the Ansab 1 find layers have been geologically sampled and documented: four field campaigns of geoscientific survey were conducted in the Wadi Sabra (2009–2012) that can now serve to contextualize the human occupation of Al-Ansab 1 (Lower Wadi Sabra) within the landscape evolution during MIS 3 and 2 [21–25].

Extensive archaeological surveys carried out from 2009–2014 in and around the Wadi Sabra detected (Fig 2) numerous Upper Palaeolithic sites. Only a few Middle Palaeolithic surface sites and many Middle Palaeolithic single finds were documented, all Middle Palaeolithic occurrences lacking stratigraphic context [26]. The surveys also yielded one Initial Upper Palaeolithic (IUP) site, embedded in redeposited sediments (Al-Ansab 2; [27, 28]), and, nearby, one early Upper Palaeolithic site, Al-Ansab 1, in primary stratigraphic context that was already known from a 1983 survey [29]. The majority of sites detected by our team belong to the Levantine Aurignacian and Late Ahmarian/Masraqan techno-cultural units, many of these sites in stratified in-situ positions [26, 29]. In the Upper Wadi Sabra, several Epipalaeolithic occupation layers and later archaeological occurrences were also discovered [see 25]. Apart from Al-Ansab 1, no other Early Ahmarian sites were found in the entire survey area.

**Fig 3:**
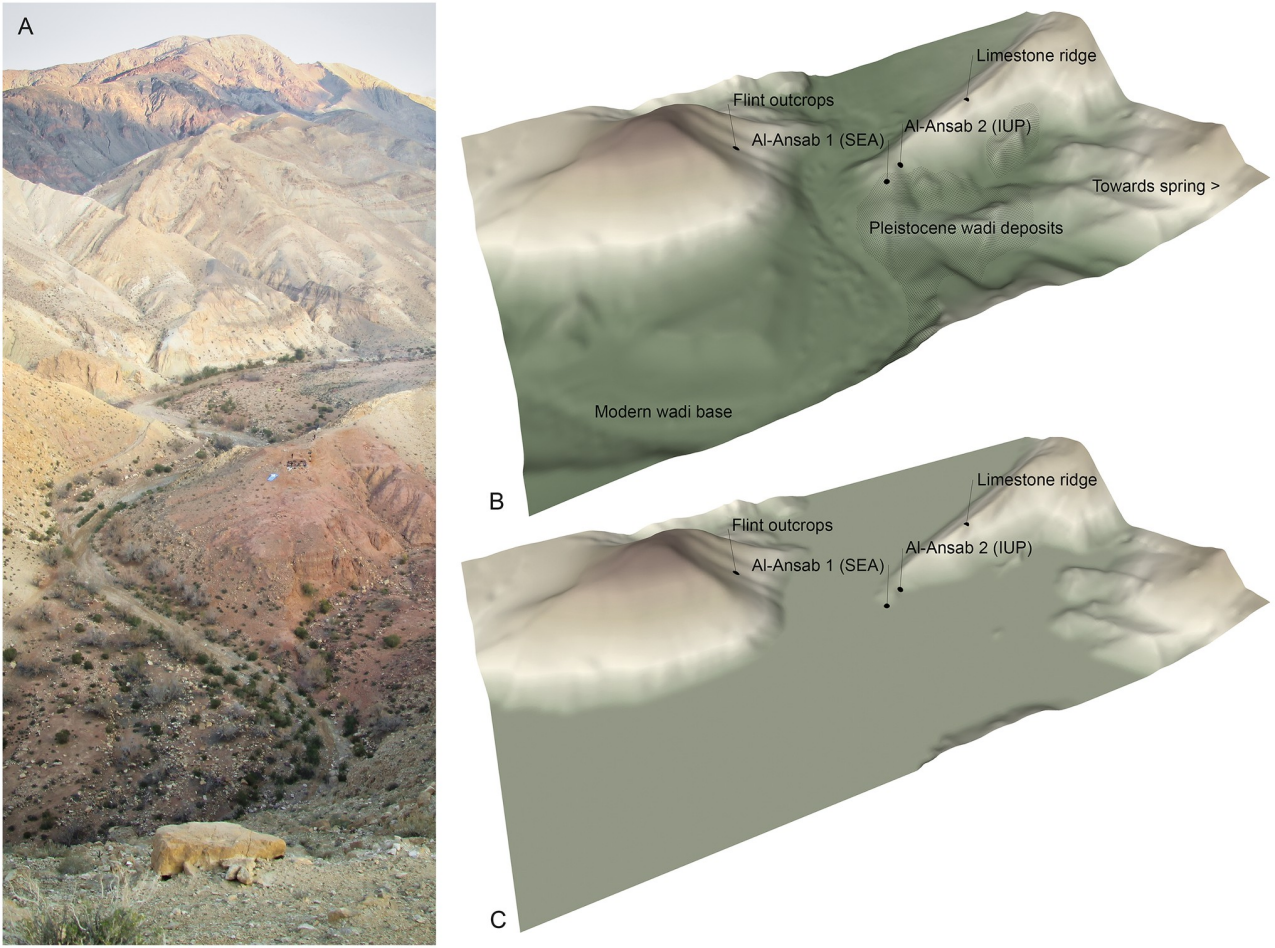
Topographic context of the Al-Ansab archaeological sites. (A) View of the Wadi Sabra from the South. Note the Al-Ansab 1 excavation site in the centre of the photograph–(B) DTM elevation image of the Al-Ansab locality (South-to-North aspect)—(C) DTM elevation model of the topographic situation around 38,000 years ago, at the time of the Al-Ansab 1 settlement site located at the fringe of a wide river floodplain (abbreviations indicate cultural attributions: SEA = „Southern Early Ahmarian”techno-cultural unit; IUP = „Initial Upper Palaeolithic”techno-cultural unit; DTM: UTM Grid 36, EPSG 32636, Coordinates: 0729360 E / 3347130 N).

At Al-Ansab 1 we discovered a well stratified Early Ahmarian occupation surface embedded in consolidated sand layers of fluvial and aeolian origin [21, 30]. Al-Ansab 1 is located in the Lower Wadi Sabra on a Pleistocene sediment promontory at 618 m asl c. 25 m above the narrow wadi bed (Fig 3). Pleistocene sediments at Al-Ansab have been preserved due to the adjacent limestone crest of the Umm Rijam Chert limestone that protects the site from erosion. The Al-Ansab 1 sediment remnant attests for the MIS 3 paleo-surface which was on a higher level in the wadi than today (Fig 3C). This indicates that the former floodplains were more extensive than today.

The surrounding ridges contain abundant flint blocks that were frequently exploited for flint knapping at Al-Ansab 1 [31]. Both the nearby flint outcrops and a spring located just a few hundred metres east of the site must have made Al-Ansab an attractive location for prehistoric hunter-gatherers (Fig 3). Since 2009, a series of field campaigns were conducted to the site [29, 21, 27, 30, 32] and sediment studies, radiometric dating and techno-typological analyses of the lithic material were carried out (see S4 Table; for definitions of artefact classes see reference section in S4 Table).

### Second research locale: Dead Sea

Vegetational remnants have not been found in the Wadi Sabra (with the exception of some charcoals). To compensate, the Dead Sea features here as a second research locale particularly contributing the reconstruction of the mid-MIS 3 vegetational record. Pollen data from the Dead Sea cores were included and analysed by the palaeobotanical research group of the CRC 806 Collaborative Research Centre [33] that is also contributing to the ICDP Dead Sea drilling programme [33, 34]. Today, the Dead Sea is bordering Israel, Jordan and the Palestinian territory West Bank occupying the lowest continental depression on the Earth (currently ca. 430 m below mean sea level (m bmsl). It is a terminal and hypersalin lake with a salinity multiple times higher than seawater (c. 27.5%) [35]. The Dead Sea is primarily fed by the perennial Jordan River but also by groundwater and several ephemeral streams experiencing occasional flash floods. It is the largest lake in the region with a surface area of about 760 km^2^ [36] and a drainage area of 42,200 km^2^ [37]. Due to tectonics in the rift valley and sea level changes, the Dead Sea region experienced profound hydrological changes over the last millions of years. After the disconnection of the Sedom lagoon from the Mediterranean Sea, the Dead Sea Basin was occupied by a series of lakes. The last interglacial precursor lake was Lake Samra, and the last glacial one was Lake Lisan [38]. The new palynological data was gained from sediment cores produced by the International Continental Scientific Drilling Program (ICDP) Dead Sea deep drilling project. The ICDP core represents the longest continuous sediment record of the southern Levant (core 5017-1-A, site 5017–1, N 3130^0^28.98^00^, E 3528^0^15.60^00^, with a total drilled length of 455.34 m).

## Methods and results

Following the previously mentioned research frame (contextual area approach) we present archaeological site distributions and new excavations at Al-Ansab 1, an Early Ahmarian open-air site (focus: Wadi Sabra near Petra (Jordan), see section „Archaeological record“). In addition, we contextualize the human occupation with the MIS 3/2 landscape evolution (focus: Wadi Sabra near Petra/Jordan, see section „Sediment record“). Further on, we combine both results with MIS 3 vegetation records from the nearest lake drilling locations available (focus: Dead Sea, see section „Palynological record“). Finally, we integrate the Dead Sea vegetation record within a regional biome model in combination with the Early Ahmarian site distribution (focus: regional, see section „Biome modeling“).

### Research permission statement

The Wadi Sabra research permit was granted 2009–2020 by the Department of Antiquities, Hashemite Kingdom of Jordan.

### Archaeological record (Al-Ansab 1, Wadi Sabra)

Al-Ansab 1 provides two Early Ahmarian strata. While the upper layer is situated at a depth of c. 2 m below surface, the lower layer appears at a depth of c. 1.45 m below the upper level. However, at the current state, only the upper layer has been excavated (24 m^2^). Hence, we solely focus on presenting the excavation results of the upper layer in this paper.

#### Excavation at Al-Ansab

**Fig 4:**
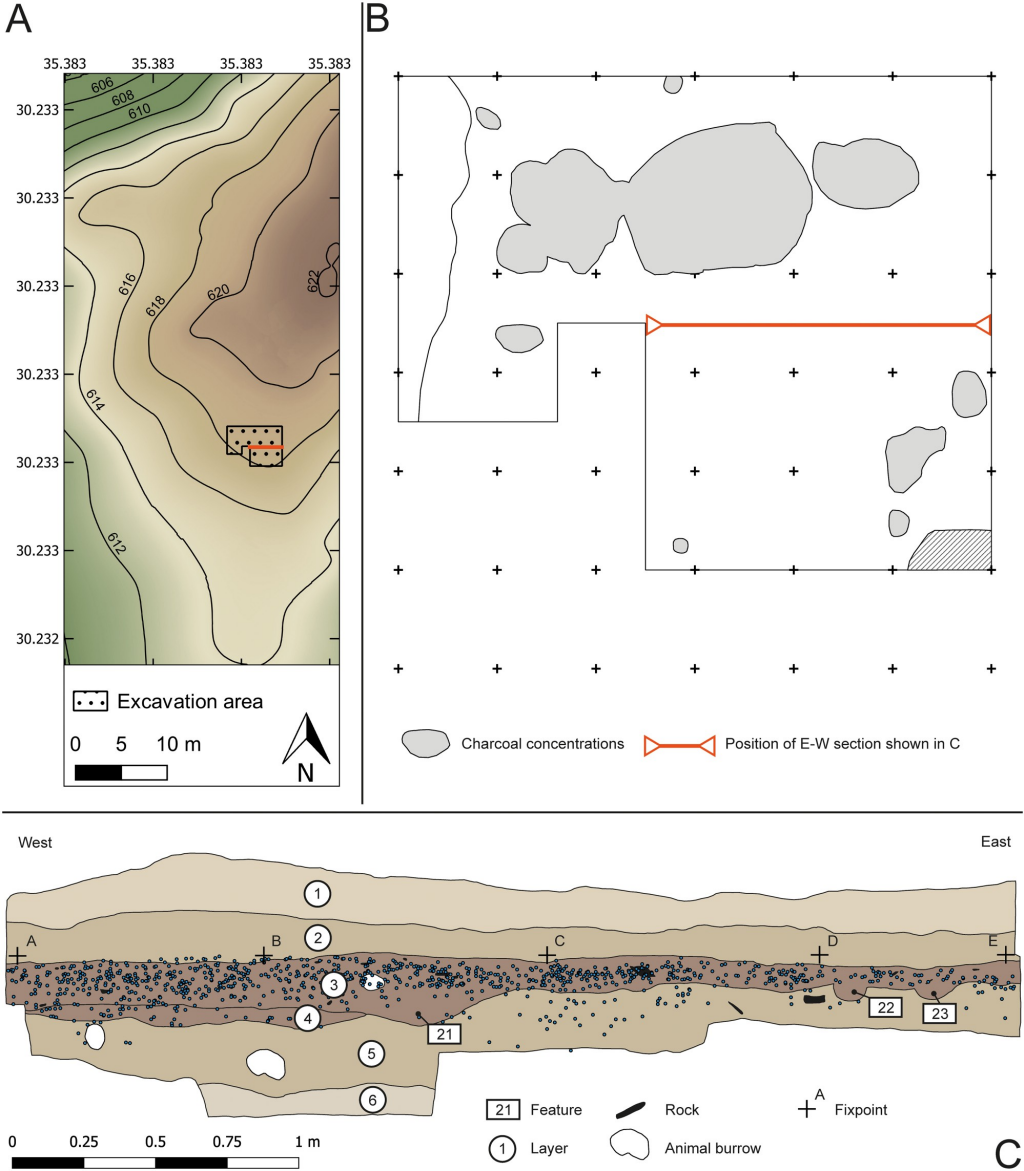
Excavation results from the Al-Ansab 1 settlement site. (A) Excavation area (shaded) located at the southern slope and close to the top of a Pleistocene sediment remnant. Red line, at northern limit of excavated area, indicates position of section below–(B) excavation area with evident features.—(C) Stratigraphic West-to-East section of the Al-Ansab 1 excavation (May 2017; see (A) for position of section)–numbering of layers: 1 fine horizontal layering of homogeneous fine sands, reddish light brown, with Calcium grains; 2 fine horizontal layering of homogeneous fine sands red-brown; 3 Al-Ansab 1 archaeological find layer (including stone artefacts, bones, ochre, charcoal etc.), of slightly consolidated fine sand, greyish light-brown, with anthropogenic features (framed numbers: 21, 22, 23), such as fireplaces and pits; 4 sediment lens of greyish light-brown fine sand with few artefacts; 5 fine horizontal layering of red fine sand, interlaced with fine gravel, quite loose, with occasional krotovinas; 6 fine horizontal layering of red, homogeneous fine sand and silt.

Excavated area and excavation technique: One-metre squares formed the basic units of the 24 m^2^ excavation grid (Fig 4A and 4B). Each square metre was further subdivided into quarter-squares measuring 50 x 50 cm each (¼ m^2^) and were then excavated in spits of 5 cm depth. All finds > 1 cm that were recovered during excavation (lithics, bones, charcoal, etc.) were continuously drawn and photographed in plan while their X, Y, Z coordinates were documented individually in a local grid system using a total station (Leica TS06ultra-2). In addition, finds > 2 cm were spatially documented with two-point measurements (e.g. blades and bladelets at their extreme axis) or more (e.g. cores, flakes, rocks, etc.) depending on the object’s contour. Finds < 1 cm were then collected and documented based on the quarter-squares and spit numbers they had been retrieved from. All sediments were screened using a sieve with 2 mm mesh size in order to facilitate near-complete recovery of all artefacts and paleoenvironmental samples.

*Site taphonomy*. The archaeological finds concentrate on a buried occupation surface constituting the upper limit of a 10 cm thick, consolidated layer of aeolian and colluvial sand (Fig 4C, layer 3). The occupation surface is almost level in E-W direction (Fig 4C) and slightly sloping down southwards. To the west, the occupation layer is limited by a buried fluvial channel with coarse sand and river gravels (Fig 4B). To the southeast, the occupation layer is cut by modern slope erosion (Fig 4B). The find-bearing layer 3 (equivalent to geological unit 1b in Fig 9B) displays a slightly grayish color compared to the underlying and covering sand layers.

**Fig 5:**
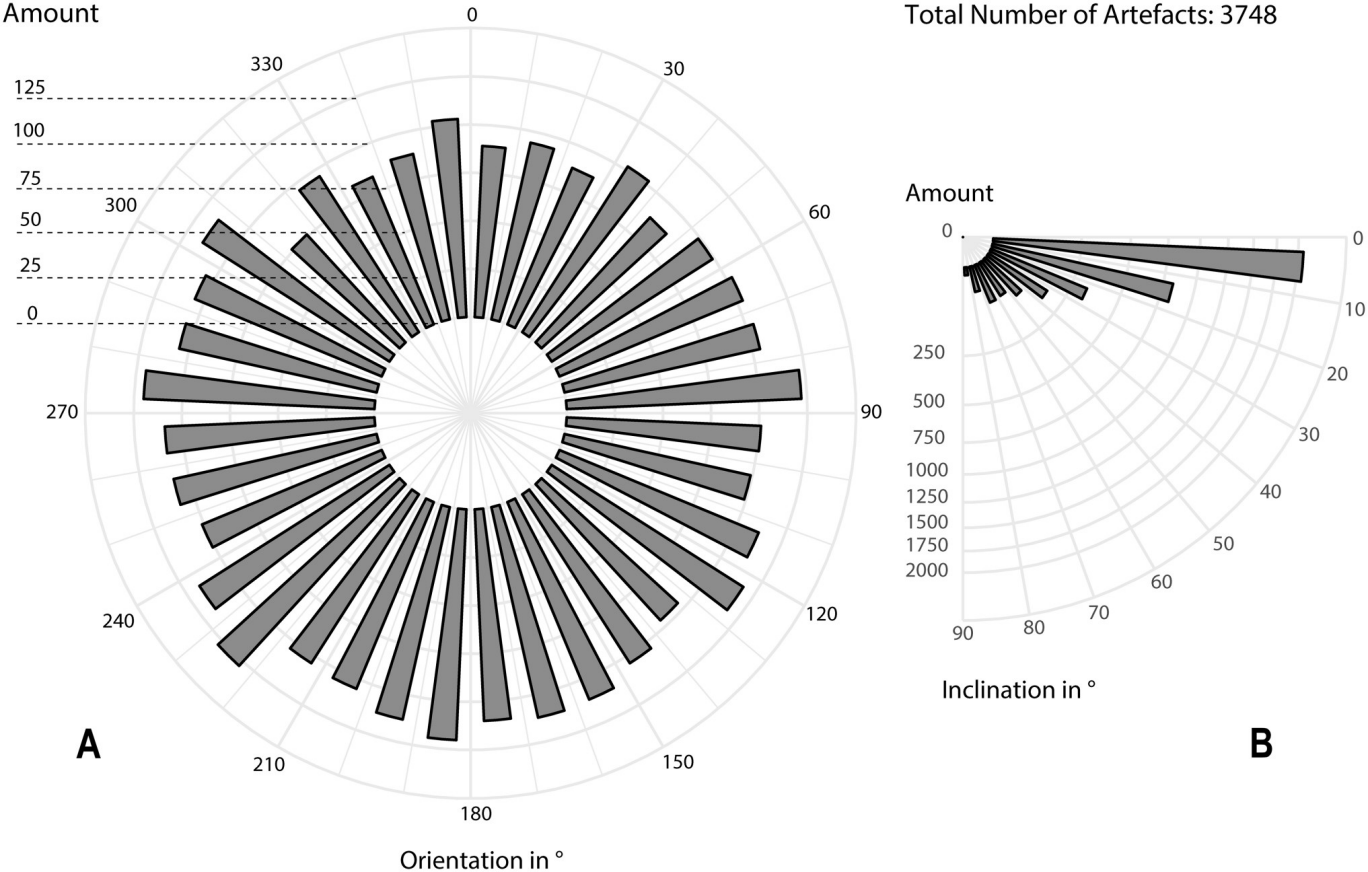
Orientation (A) and inclination (B) of elongated lithic artefacts. Each bar shows the absolute number of artefacts within the given orientation and distance-class (by 10° steps). The diagram shows all elongated lithic artefacts that were measured with two points during excavation (Total n = 3748; software: ggplot).

Orientation of elongated finds (Fig 5A) is almost randomly distributed throughout all compass directions and the position of the finds was documented as mostly horizontal at the moment of excavation Fig 5B). Three-dimensional positioning and vertical mapping of archaeological finds confirms the excellent preservation of the find layer (Fig 4B and 4C). The vertical distribution of finds is, in line with the occupation surface, sharply limited to the overlying sand layer (Fig 4C). By contrast, some artefacts proliferate into the underlying sediment, following the shape of some shallow ashy depressions (cf. Fig 4B). So far, taphonomic analysis of the central part of the occupation surface has disproven any post-sedimentary redeposition of artefacts, indicating, in conventional terms, an in-situ preservation.

**Fig 6:**
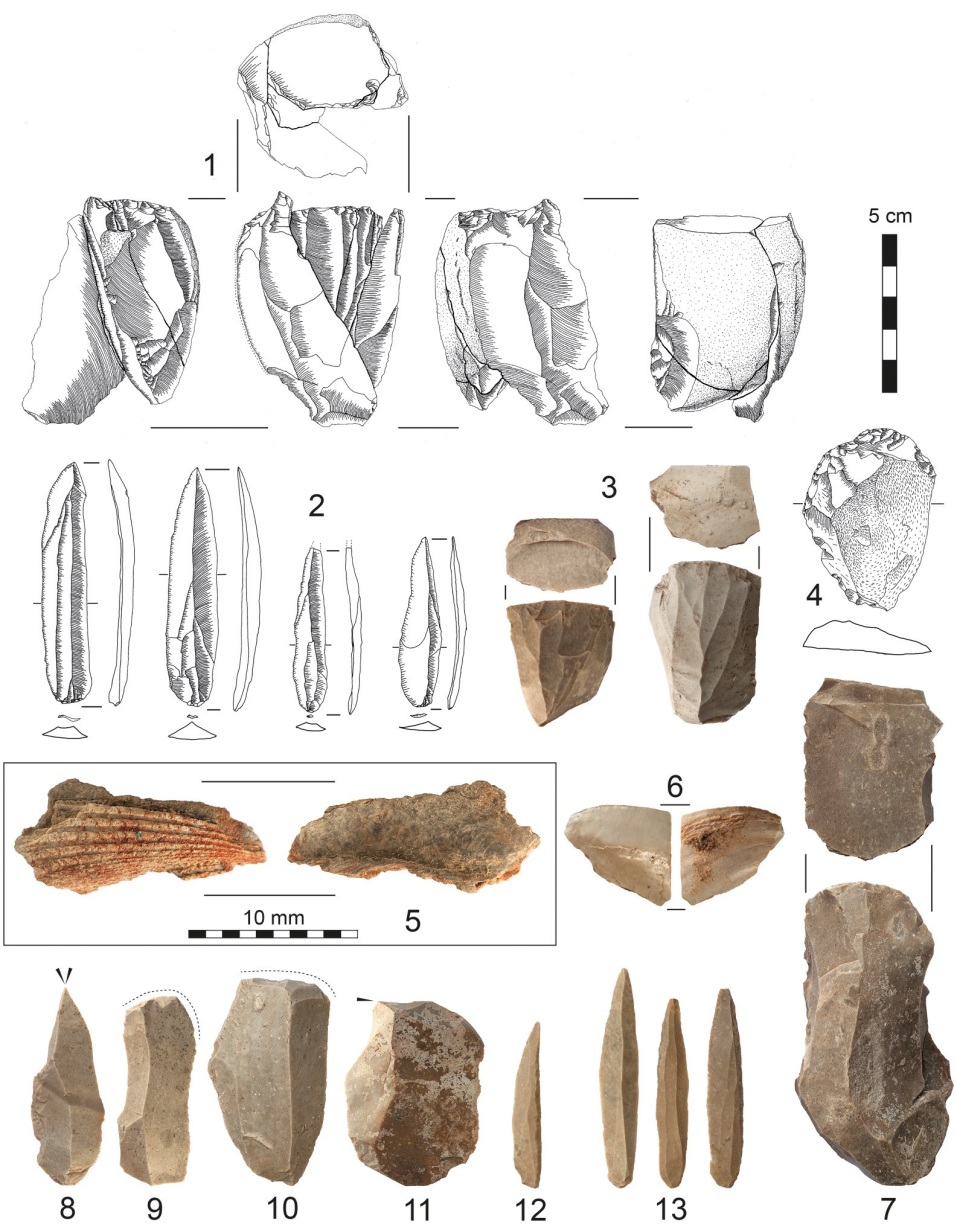
Artefacts from Al-Ansab 1. (1) Refitted blade core, (2) El-Wad points (3) blade cores, (4) end-scraper, (5) marine shell fragment with ochre staining, (6) marine shell fragment, (7) blade core (8) dihedral burin, (9, 10) end-scrapers, (11) burin and (12, 13) El-Wad points.

*Archaeological finds*. The majority of finds is comprised of lithic artefacts (N = 40,491), followed by faunal remains (n = 1651) and ochre pieces (n = 211) (Fig 6, Table 1). Poor bone preservation is reflected by small-sized fragments and heavily weathered surfaces. Throughout the find layer, ochre occurs frequently, often correlating with concentrations of modified lithics. We retrieved 92 charcoal and 50 sediment samples, the latter including samples for Optically Stimulated Luminescence (OSL) dating and micromorphology.

**Table 1:** Al-Ansab–1—Inventory of excavated finds (2015–2018).

		N	Relative frequency
**General material categories**	Flint	40491	94.0%
	Non-flint Rocks	465	1.1%
	Charcoal pieces	234	0.5%
	Ochre pieces	211	0.5%
	Faunal remains	1651	3.8%
	Other materials	42	0.1%
**Cores and debitage**	Cores	233	0.6%
	Flakes	9385	23.2%
	Blades	3096	7.6%
	Bladelets (width <1.2 cm)	7750	19.1%
	Chunks	20027	49.5%
	Total (excl. chips)	40491	100.0%
	Chips (< 1cm)	more than 1.6 kg	
*Total number of tools in assemblage*		*244*	*0*.*6%*
**Tool types**	El-Wad points	88	36.1%
	Burins	47	19.3%
	End-scrapers	37	15.2%
	Notched/denticulates	9	3.7%
	Edge retouched pieces	35	14.3%
	Burin spalls	28	11.5%
	Total	244	100.0%

**Fig 7:**
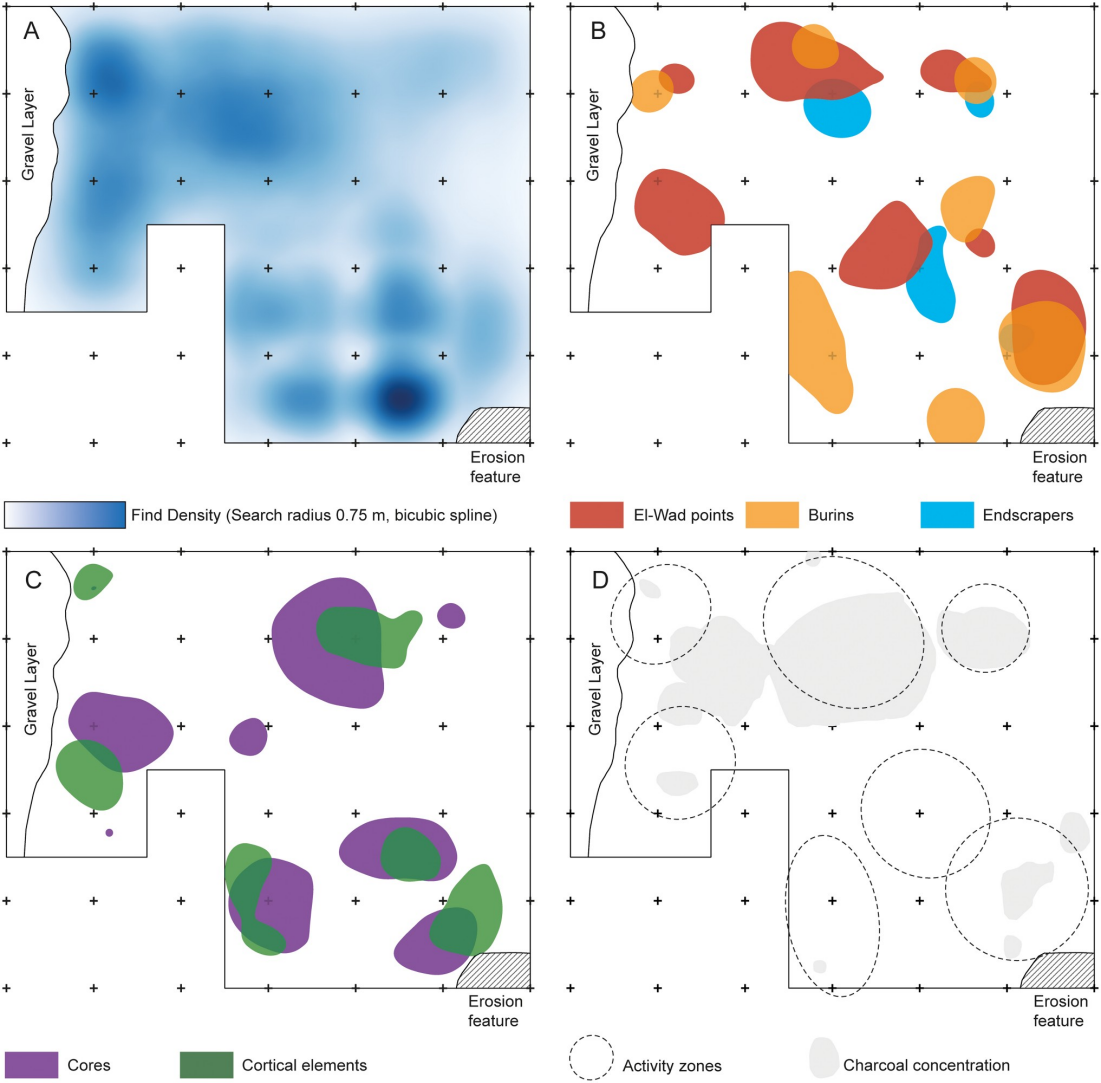
Distribution maps of different artefact classes in Al-Ansab 1. (A) General interpolated find density in the excavation area (B) Clusters of different tool types in the excavation area (C) Clusters of cores and cortical elements in the excavation area (D) Charcoal concentrations and delineated activity zones. Note that remnants of blank production and tool use (A, B, C) each scatter around to the same centres of activity (D) thus indicating 7 events of activity, all producing similar assemblages. Spatial analysis was performed with the Heatmap tool in QGIS 2.18. Clusters were identified based on selected isolines from computed kernel density estimates. Spatial data covers the field campaigns from 2015 to 2018.

*Intra-site patterns*. The general distribution of finds (Fig 7) reveals an internal pattern of the occupation surface, with seven separate artefact scatters of approximately one square metre each (Fig 7D). The content of all scatters is similar, with all artefact classes and tool types present in each single scatter (Fig 7B and 7C). Only occasionally such scatters appear to be connected to ashy lenses, possibly the remnants of fireplaces (Fig 7D). The southern part of the excavation area is dominated by a particularly large ash concentration.

**Fig 8:**
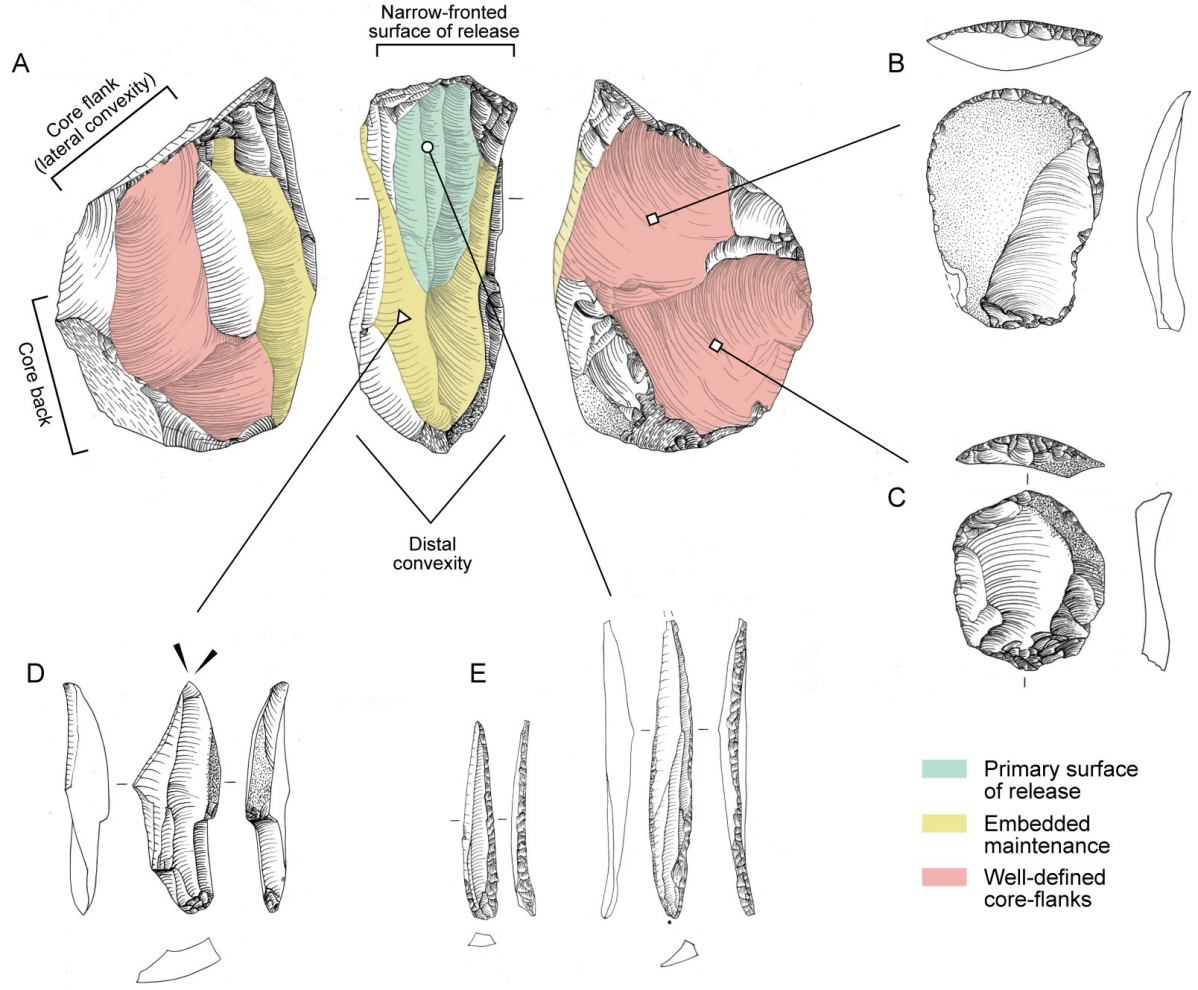
Technological nature of artefacts from Al-Ansab 1. (A) Conceptual division of core exploitation surface into core back (left), primary surface of blade/bladelet release (centre) and two core flanks (left and right); (B) Cortical flake from initial core flank preparation transformed into tool (end-scraper); (C) Flake with small portion of cortex from secondary core flank preparation transformed into tool (end-scraper); (D) Blade from embedded maintenance (yellow shading) in order to reshape the primary debitage surface of blade/bladelet release (green shading), subsequently transformed into tool (dihedral burin); (E) Pointed bladelets from primary debitage surface (green shading) transformed into typical El-Wad points. Relations are idealized and not based on physical refits but rather grounded on the reconstruction of the early Ahmarian technological system [27]. Lithic artefacts are not to scale.

*Lithic artefact production*. When assessing the relative proportions of various artefact classes, cores (0.6%) and tools (0.6%) are rare, while blanks (50%), chunks (49.5%) and chips are abundant which is in line with the interpretation that the Al-Ansab 1 camp site would include a primary workshop component. This culminates in a high blank-to-core ratio (86.8:1), a high blank-to-tool ratio (82.9:1) and a high debris-to-core ratio (86:1) while the tool-to-core ratio is low (1:1). The lithic material recovered from Al-Ansab 1 represents the entire chaîne opératoire (Fig 8) from core initialisation to re-tooling as evidenced by the presence of cortical pieces (>50% cortex cover), core trimming elements (CTEs), flakes, blades, chips (flakes <1 cm), cores and tools (cf. S4 Table). Common CTEs are crested blades and core tablets indicating the core initialisation and preparation methods were conducted at Al-Ansab 1. Cores belong to the sub-pyramidal type and were exploited in a frontal, unidirectional manner [6]. Their extraction surfaces largely show blade and bladelet scars.

*Broken artefacts and burnt pieces*. Fragmentation of artefacts affects almost half of all lithic finds (45.1%, see [29, tab VI-13]). The abundance of broken artefacts could be the result of trampling during intensive and repeated periods of site occupation. Traces of burning occur among c. 10% of all single plotted lithics. While chunks frequently show traces of burning or are shattered (33%), other artefact classes—bladelets in particular—rarely show signs of heating (5%).

*Lithic toolkit*. The toolkit is mainly comprised of burins (19.3%), end-scrapers (15.2%) and El-Wad points (36.1%; c.f. Table 1) which identifies Al-Ansab 1 as a typical Southern Early Ahmarian assemblage with diagnostic lithic artefacts such as El-Wad points, abundant narrow bladelets and unidirectional bladelet cores.

*Organic finds*. In addition to the mentioned finds, two marine shell fragments were retrieved from the cultural layer (Fig 6). One of the shells is stained with ochre. Both specimens are fragmented and show no preserved signs of perforation indicating use as ornaments [39, 40]. While both fragments can be classified as marine bivalve Molluscs, their species membership could not be determined, yet. However, considering the ochre-stained shell, an attribution to the Cardiidae family (e.g. *Glycimeris* sp., *Acanthocardia* sp.) is possible, while the other specimen might belong to *Panopea* sp. instead. The same range of bivalve Molluscs has been reported from broadly contemporaneous sites along the Mediterranean coast [40–42]. The Al-Ansab 1 Molluscs must have been transported to the site by humans either from the Red Sea or the Mediterranean, which would mean a minimum transport distance of 90 km from the Red Sea and even 170 km from the Mediterranean. The faunal assemblage recovered is small and poorly preserved. The majority of bones are only preserved as splinters and small fragments limiting the exact determination of fauna present at the site. While most of material is not yet analysed, 143 bone fragments were investigated. To date, only gazelle could securely be established at Al-Ansab, based on several molars [31]. Gazelle likely represents hunted prey exploited at the site.

*Overall excavation result*. Based on techno-typological classification, Al-Ansab 1 is a typical Southern Early Ahmarian site, characterised by narrow blades and bladelets production removed from unidirectional volumetric cores and (bladelets) from carinated pieces [27]. These blanks were then retouched, producing El-Wad points, edge-retouched pieces and typical Upper Palaeolithic tools [2, 4, 5, 29]. The site plan (Fig 7) argues for the repetition of the same spectrum of activities within each of 7 artefact scatters, rather than different activity zones mirrored by distinct clusters of specific components. Accordingly, each concentration is to be interpreted as a similar remnant of a short occupation phase, with hunting related tools (El-Wad points) and maintenance tools (end-scrapers and burins) indicating different kinds of activities present. Based on a small number of faunal remains, gazelle featured as a hunted prey. Sea shell fragments indicate connections to the coast of the Mediterranean or the Red Sea.

#### Radiometric dating of the human occupation

To determine an absolute age estimate for the archaeological layers at Al-Ansab 1, optically stimulated luminescence (OSL) dates and ^14^C dates were obtained. Luminescence dating results provided a relatively broad temporal classification between 45 and 32 ka BP [30], indicating a relatively short period of sedimentation for the existing 20 m of sandy deposits. Radiocarbon dating performed on charcoal found at the Al-Ansab 1 cultural layer allowed to independently test and enhance temporal resolution. All seven ^14^C samples were pretreated and measured at the CologneAMS centre. Pretreatment techniques followed the AAA (Acid-Alkali-Acid) protocol (see [43] for detailed description of Cologne lab procedures). Due to limited sample volumes at Al-Ansab 1, the Acid-Base-Oxidation (ABOx) protocol, which has occasionally delivered older dates than competitive protocols [44, 45], was not applied because ABOx would have enhanced the risk of sample volumes loss of Al-Ansab 1 that in turn may have increased dating uncertainties (for detailed comparison of ABOx and competitive protocols see Wang in [46]; for recent criticism of ABOx see e.g. Rebollo [47] and Alex [11]). All samples underwent combustion and graphitisation (Automatic Graphitisation Equipment linked to an Elementar VarioMicroCube element analyser with a combustion tube and a reduction tube). Measuring was carried out at the 6 MV Tandetron AMS established 2010 at the CologneAMS centre [48]. Data quality is assured by parallel measurement of organic and bone standard materials from the Fifth Radiocarbon Intercomparison exercise [43].

Seven ^14^C dates were obtained on charcoal samples in several locations in the excavated area (Table 2). The dates show a high level of concordance placing the occupation of Al-Ansab 1, layer 1 between 36,963 **±** 610 and 37,890 ± 630 (cal BP). Both OSL and ^14^C results overlap within the 2σ range (Fig 14; see S3 Table for complete list of Early Ahmarian ^14^C dates).

**Table 2:** ^14^C dates from Al-Ansab 1.

Lab. No.	ID	Date	Location	material	Conv. Age (yrs BP)	+/-	δ^13^C (‰)	C weight (mg)	Pre-treatment	Cal BP (1σ)	±	Cal BP (2σ)	±
COL #													
2475.1.1	AN001	2013	N-S Trench, layer 2/3 (Fig 4D)*	charcoal	32869	409	-27.4	1.00	AAA	36963	610	37182	1105
2476.1.1	AN002	2013	168–17, layer 3 (Fig 4D)*	charcoal	33041	419	-26.3	1.00	AAA	37279	633	37316	1083
2477.1.1	AN003	2011	184A, Pos. 3, layer 3 (Fig 4D)*	charcoal	33292	432	-25.7	1.00	AAA	37570	640	37494	1093
2478.1.1	AN004	2011	184A, Pos. 2, layer 3 (Fig 4D)*	charcoal	33564	444	-27.7	0.99	AAA	37890	630	37741	1167
2479.1.1	AN005	2013	N-S Trench**	charcoal	33552	460	-28.9	0.51	AAA	37864	648	37730	1197
2480.1.1	AN006	2013	N-S Trench**	charcoal	32937	439	-39.1	0.44	AAA	37143	644	37231	1138
2481.1.1	AN007	2013	N-S Trench**	charcoal	33447	440	-23.2	1.00	AAA	37753	634	37623	1130

**Fig 9:**
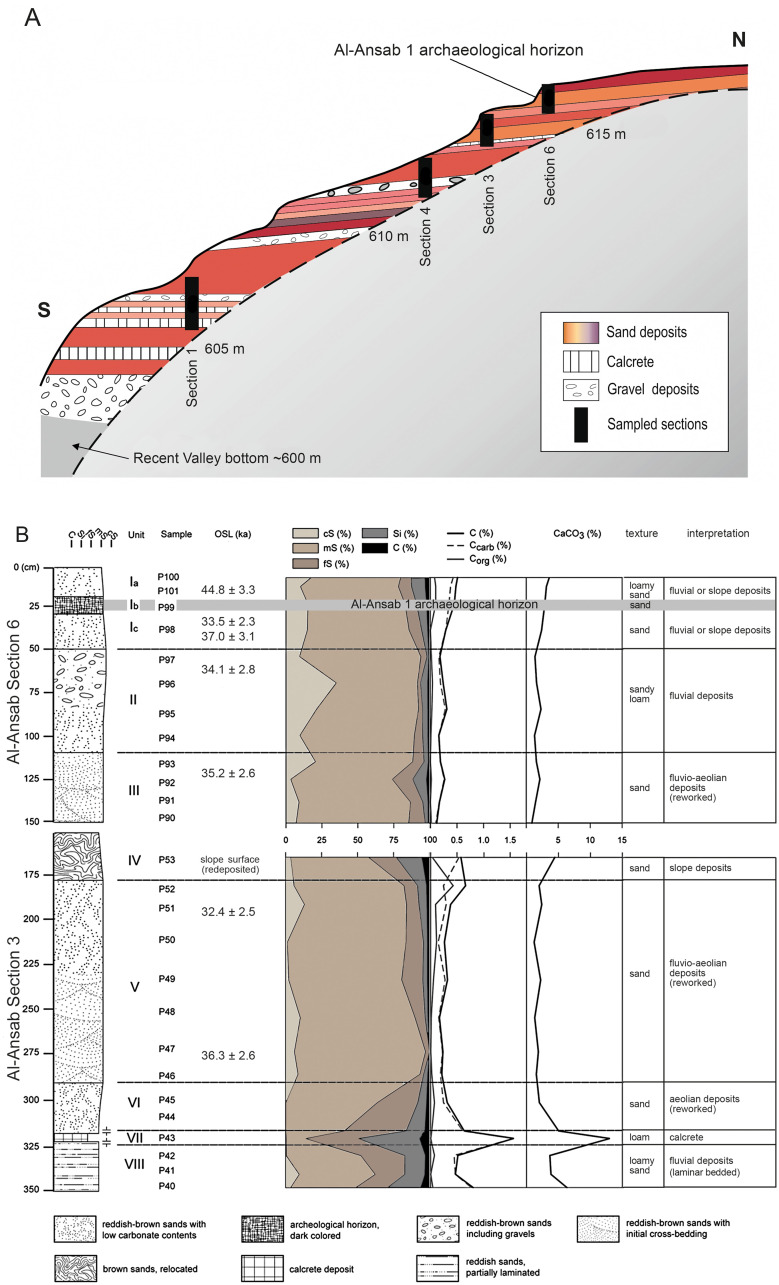
Local geological sequence of the pleistocene wadi fill around the Al-Ansab 1 Early Ahmarian settlement site. (A) South-to-North schematic section through the Pleistocene sediment remnant bearing the Al-Ansab 1 site, from the base of the present Wadi Sabra dry-river channel (S = 35.3828 E / 30.2320 N) to the top of the wadi fill (N = 35.3829 E / 30.2329 N). Note position of Al-Ansab 1 archaeological horizon and related geological section described below; (B) Selected data resulting from sediment analysis of geological sections 6 and 3 (see Fig 7A). Proxy data attest to calm accumulation of eolian and fluvial sands, the archaeological horizon connected with initial soil formation and slightly consolidated surface (for details and statistical measures see [21]. Note that stratigraphic resolution is higher than radiometric resolution achieved by OSL dates, as OSL values indicate an approximate age range of 35 +/- 3000 ka without exact match between mean ages and stratigraphic positions. OSL data from CRC 806 [30].

Calibration was conducted using the Intcal13 calibration curve provided by the OxCal 4.3 software package (* sample taken from principal archaeological horizon attributed to the Early Ahmarian; ** sample taken from profile section of un-excavated, lower archaeological horizon matching with upper part of geological unit III in Fig 9B). ^14^C measurements provided by CologneAMS. AAA = Acid-Alkali-Acid.

### Geological record (Wadi Sabra)

Compared to the combined Upper and Lower Wadi Sabra sequences, only the earlier part of the sedimentary sequence is to be found in the Lower Wadi Sabra. Mapping of ASTER and SPOT-5 satellite images showed a link between Upper Palaeolithic occurrences and reddish Pleistocene wadi deposits [49]. Results from sedimentological and geochemical analysis supported by archaeological and radiometric dating provide evidence for fluvial and fluvio-aeolian sedimentation during MIS 3 and 2 from 45 ka to 18 ka, followed by at least one erosional event that finally determined the recent valley morphology [22, 23]. Several calcretes, buried surfaces and initial soil horizons indicate phases of landscape stability and more humid climatic conditions comparing to the modern climate. Fig 9 compiles the different sections and the wadi profile with special focus on the Al-Ansab section in the Lower Wadi Sabra.

#### Description of the Al-Ansab sections

115 samples from the upper and the lower Wadi Sabra were analysed by M. Bertrams [21–23] in terms of geochemical and physical attributes including silicon and carbonate contents (C, Ccarb, Corg and CaCO3 values) and grain characteristics (particle diameter, mean, sorting, kurtosis and skewness; details of sample preparation and analysis [21]). At Al-Ansab (Fig 9A), the basal part of the wadi fill contains several layers of coarse grained and poorly sorted wadi-sediments. This reworked mixture of local rocks like quartz, sandstone, limestone and metamorphic fragments indicate high energy fluvial deposition or debris-flow events of unknown age and duration (Fig 9B, Unit VIII). The investigated sediment sections above the gravels mainly comprise reddish-brown sands, intercalated by fine-grained calcrete laminations indicating a less energetic fluvio-aeolian or sheet-flow deposition (Units VII–III). Within these units, gravels are mostly absent, and the deposits consist of moderately to poorly sorted sands and loamy sands with particular laminations and crossbedding structures which indicate aeolian influence. At the top of the Al-Ansab stratigraphy, an increase of the fine fraction in the sediments can be observed from unit II to the Ahmarian layers in unit I. However, the sandy loam and gravels of unit II, the sand-matrix in units Ib and Ic, and the loamy sand in unit Ia, generally reflect fluvial or slope deposits including an aeolian component [22].

As a result, the deposits provide indicators for multiple phases of positive sediment budget during the first half of MIS 3 possibly connected with somewhat more humid climate than today (Table 3). Generally, the sediments were deposited in a low energy fluvial context of migrating, shallow streamlets, only sometimes interrupted by fluvial discharge events reflecting a pronounced seasonality and a potentially disturbed vegetation cover [24]. The latter are the prevailing erosional agents typical for wadis in the study area today. It has to be stressed that these sedimentological archives provide information on the development of environmental conditions on a local scale while the regional and supraregional perspective is covered by the palynological data, which will be presented in the subsequent section.

**Table 3:** Overview summarizing the sediment and settlement history of the Upper and Lower Wadi Sabra combined.

Timeframe	Sediment history	Human occupation
15 ka BP and later	Stopping of sedimentation, increasing erosional intensity, erosion prevails over accumulation	Human occupation in the Upper Wadi Sabra, preserved only as surface sites on 15 ka consolidated surface
20–15 ka BP	Wadi sediments interrupted by fluvial or „flash flood“-horizons	Human occupation in the Upper Wadi Sabra, preserved as stratified horizons within sediment sequence
45–20 ka BP	Wadi sediments mostly resulting from low-energy deposition	Human occupation between 45 and 38 ka BP in the Lower Wadi Sabra and from 30 ka onwards in the Upper Wadi Sabra, preserved as stratified horizons within sediment sequence
before 45 ka BP	Gravels indicate high-energy fluvial events	Middle Palaeolithic surface finds, no archaeological sites preserved

Note the gap in human occupation between 38 ka and 30 ka BP.

#### OSL dating of sediments

In addition to sedimentological analysis, OSL dating was applied to sediments in the Wadi Sabra region. Dating the fluvial and colluvial samples was challenging due to the fact that the samples showed low quartz luminescence sensitivity. Moreover, partial bleaching of the deposits was expected prior to deposition [30]. The luminescence data from the site of Al-Ansab provide evidence of a phase of continuous aggradation active from 45 ka onwards [30], leaving more or less homogenous fluvial and fluvio-aeolian sand deposits. These sediments are interpreted by Bertrams et al. [21] to be the result of a regional climatic regime inducing higher sediment supply and lower runoff intensities than today. At this time, the onset of the Upper Palaeolithic occupation of the Wadi Sabra is marked by the Al-Ansab 1 archaeological site. To the north of the Al-Ansab 1 site and in the upper Wadi Sabra, these homogenous deposits are followed by a series of unconformable soil horizons, indicating periods of stable surfaces, probably postdating 30 ka and extending until the end of the Last Glacial Maximum (LGM). Whereas sedimentation came to a halt around 32 ka BP in the lower Wadi Sabra (including the Al-Ansab 1 area), accumulation of wadi sediments continued in the upper Wadi Sabra until the Early Natufian, as evidenced by small artefact bearing sediment remnants high above the recent valley floor and close to the sandstone cliffs delineating the Wadi Sabra.

### Palynological record (Dead Sea)

More than 250 m of the Dead Sea 5017-1-A sediment core corresponding to the last interglacial-glacial cycle were recently palynologically investigated by two of the present authors [33, 34]. The sediment depth of the analysed samples encompasses 199.07–92.35 m. The investigated interval belongs to the Lisan Formation. 76% of the Lisan Formation of the 5017-1-A core are mass transport deposits, showing disturbed, slumped, and homogenous sediment sections. The remaining sediments are composed of laminated alternating aragonite and detritus (aad facies) with some gypsum laminae. We only sampled the latter facies, resulting in sampling gaps of different length. Pollen preparation of 203 sediment samples with a sample volume of mostly 4–6 cm^3^ was processed following a standard protocol (see [33] for detailed protocol description). At least 500 terrestrial pollen grains were counted in each sample. Obligate aquatic plants were not included in the terrestrial pollen sum to exclude local taxa growing in the lake. Furthermore, destroyed and unknown pollen were excluded from the terrestrial pollen sum, which was used to calculate percentages of the pollen assemblage.

#### Main characteristics of the pollen sequence

**Fig 10:**
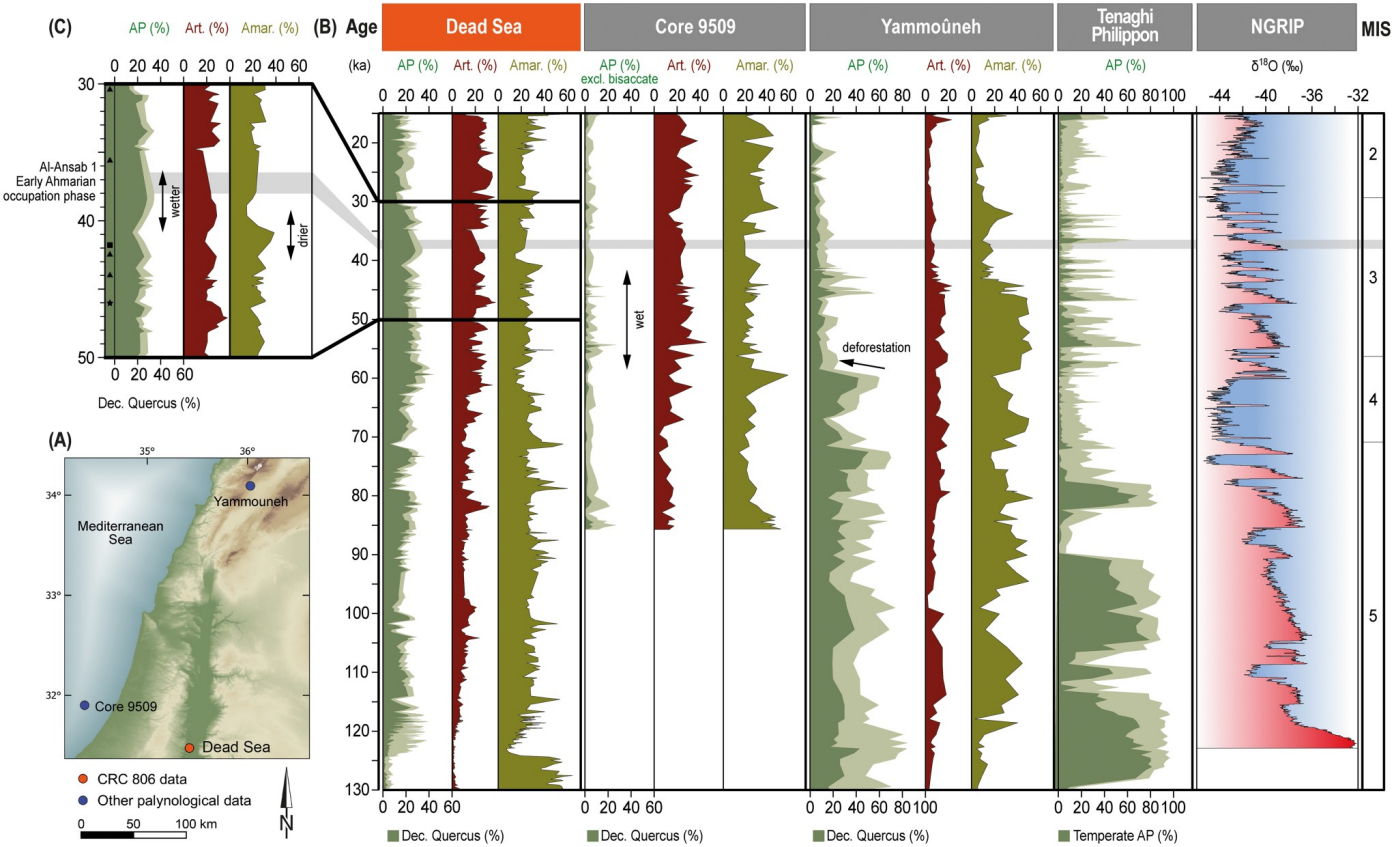
Regional comparison of Levantine vegetation sequences and reference records. (A) Levantine drilling locations. Coordinate System used: WGS 84/EPSG 4326. (B) Comparative chronological sequence of selected taxa from Dead Sea (ICDP core 5017-1-A) [33, 34], Mediterranean Sea (core 9509) [50] and Lebanon (Yammouneh) [51]. AP = arboreal pollen, Art. = *Artemisia*, Amar. = Amaranthaceae. Note that bisaccate pollen are greatly overrepresented in marine sediments. Vegetation data from Tenaghi Philippon (Greece) [52, 53], oxygen isotope data from Greenland ice shield (North Greenland Ice Core Project [54] and marine isotope stages (MIS) [55] added for reference. The Levantine vegetation data indicate cyclic, but slight variations of the vegetation cover during MIS 3 (57,000 B.P. to 29,000 B.P.) that coincide roughly with Dansgaard-Oeschger cycles. Due to the low variations, nutritional effects on grazing ungulates (gazelle etc.) would have been of marginal importance. Superimposed on this low amplitude cyclicity, a general trend towards a slow extent of open *Artemisia* steppe in the southern Levant within MIS 3 is visible. In the northern Levant, the landscape remains open and dry, while probably local preservation conditions lead to changes in the pollen assemblage. (C) Blow-up of Dead Sea pollen curves with available age determinations (rectangle = radiocarbon date, square = Uranium-Thorium date, star = correlated Uranium-Thorium date) after [33].

The last interglacial was initiated by a warm and dry phase leading to an open landscape with sparse vegetation cover. Amaranthaceae show a long-lasting maximum (Fig 10). This phase was followed by the last interglacial optimum going along with a pronounced environmental change. Drought-adapted trees and shrubs particularly olive trees and *Phillyrea* spread suggesting seasonally more available moisture. The desert belt was forced back towards the South and East [34].

During the last glacial (MIS 5d–2), seasonality was less extreme as suggested by the retreat of sclerophyllous trees. The amount of trees and shrubs–mainly deciduous oak and juniper/cypress–largely corresponded to the available water for plants. Short intervals of low AP values and high amaranth values indicate arid phases. *Artemisia* steppe gradually increased during the last glacial probably indicating a long-lasting cooling trend. It reaches maximum values during MIS 2. Together with the virtual absence of frost-sensitive pistachio, this suggests that MIS 2 was the coldest phase of the investigated timeframe [33].

As a result, environmental conditions in the Dead Sea region during MIS 3 were generally diverse and stable. The landscape was formed by a mix of steppe, desert and woodland vegetation offering a large variety of habitats for animals and humans. Although small changes among these vegetation types occurred through time, changes were of rather small nature compared to previous times and other records. Dry phases were not as pronounced as during MIS 5/4 allowing constantly sufficient water for plants [33]. However, on a smaller scale, some variations become apparent. Most noticeable is a drier period marked by an Amaranthaceae peak followed by a wetter period marked by an increase of trees and shrubs. Despite several chronological markers of the Dead Sea record (Fig 10), the exact dating of these climate variations is challenging. According to previous studies [e.g. 56, 57], North Atlantic Heinrich events were expressed as dry and cold phases in the Dead Sea region. Thus, the described dry phase might correlate to H4 dated to ca. 40–38 ka BP [15]. In any case, the Al-Ansab 1 Early Ahmarian occupation phase took place after this dry period during a slightly wetter phase, matching the GIS 8 warm interval.

### Biome modeling

#### Vegetation types observed

The palynological results indicate the occurrence of Mediterranean woodland, Irano-Turanian steppe, and Saharo-Arabian desert vegetation. The abundance of these vegetation types changed through time in response to changing climate conditions (Fig 10). Arboreal pollen (AP) are mainly composed of deciduous oak (dec. *Quercus*), juniper/cypress and sclerophyllous trees like olive tree. Thus, they overall represent the Mediterranean biome, which is found in areas with at least 300 mm annual precipitation. Wormwood (*Artemisia*) is the main constituent of the Irano-Turanian biome and is adapted to annual precipitation rates of ca. 100–300 mm [58]. Amaranths (Amaranthaceae incl. the former Chenopodiaceae family) are main representatives of the Saharo-Arabian desert biome receiving nowadays annual precipitation rates below 100 mm [36, 58].

#### Distribution analysis of biomes and archaeological sites

**Fig 11:**
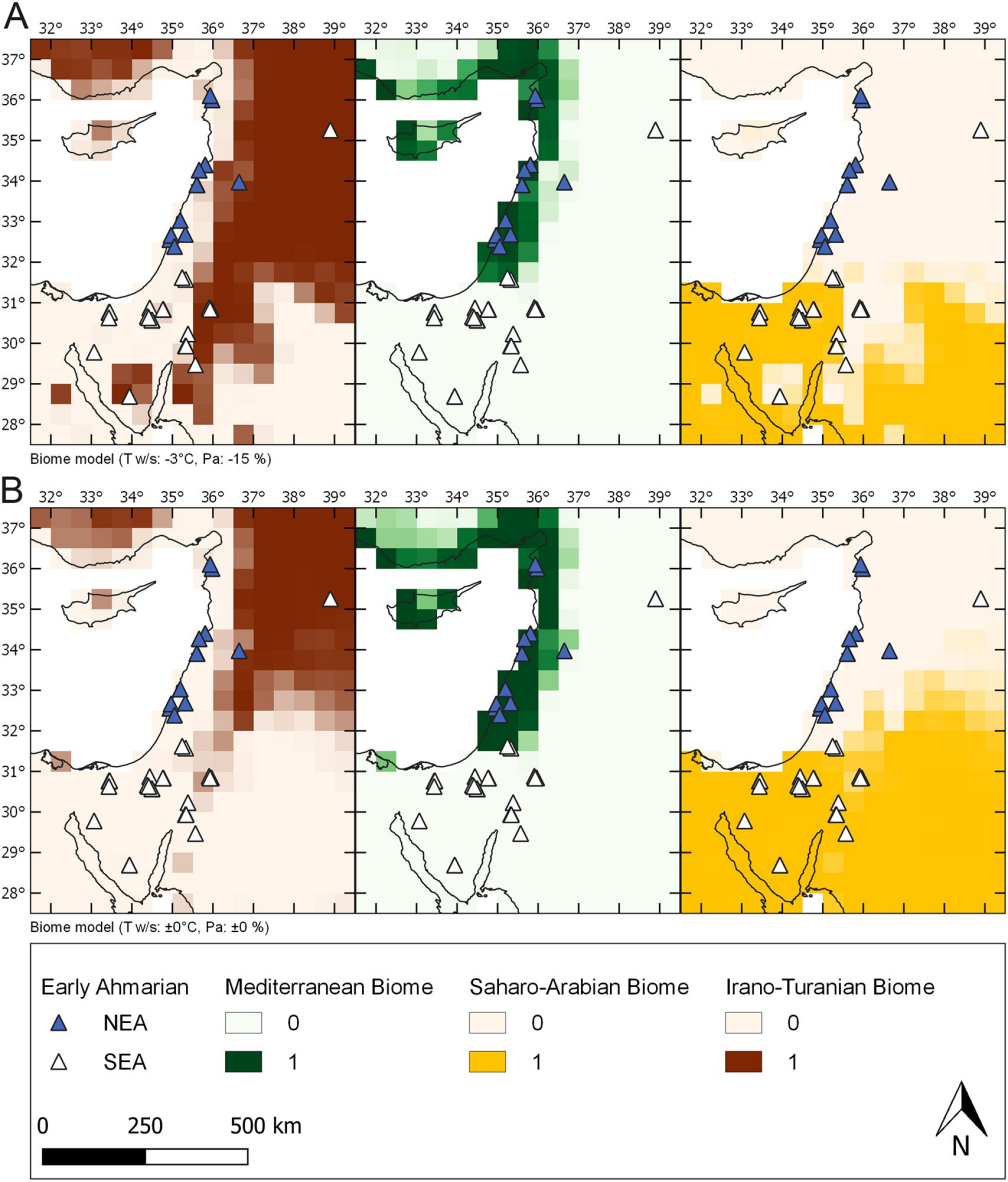
Biome distribution of the Levant with archaeological sites attributed to the Early Ahmarian cultural unit. (A) Probable MIS 3 biome distribution relevant for the time range of the Al-Ansab 1 human occupation around 39–37 ka BP with a reduced summer and winter temperature (TW/S) of 3°C and a reduced annual precipitation (PA) of 15%. (B) Modern biome distribution with unchanged climate parameters. Colours indicate share of vegetation connected with the Irano-Turanian biome (steppe), the Mediterranean biome (woodland) and the Saharo-Arabian biome (desert and semi-desert) [modified after 33]. Triangles indicate archaeological sites according to Fig 1, above. The MIS 3 model depicts the establishment of a steppe corridor through the Levant. Coordinate System used: WGS 84/EPSG 4326.

As the pollen sequence from the Dead Sea, along with sections from Lebanon, the Mediterranean, document local pollen input into sediment volumes biased by preservation, pollen productivity and transport of plant species etc. [33], spatial modeling is needed to apply the vegetational sequences to larger portions of land surface. Here we use a biome model (Fig 11) based on the modern distribution of three participating biomes, the Mediterranean biome, the Irano-Turanian biome and the Saharo-Arabian biome [33]. The model is designed to deliver biome distributions derived from different climate scenarios, according to variations in PA (annual precipitation sum) and TW/S (mean summer and winter temperature). Fig 11 shows one selected model output (Fig 11A) out of 4 alternatives originally modeled (cf [33] Fig 4) along with the model set up for modern PA and TW/S. Comparison of the 4 alternative model outputs with MIS 3 Levantine vegetation records allowed for evaluation of the modeled climate scenarios [33]. As a result, (cf [33] 108–109) the pollen signal from the MIS 3 section of the 5017-1-A Dead Sea core matches the first scenario (Fig 11A) due to high proportions of Irano-Turanian taxa and moderate amounts of Mediterranean taxa. The model indicates that a small reduction in precipitation (15% less than modern PA) along with temperatures reduced by 3°C (compared to modern TW/S) does still allow sufficient moisture availability for the growth of Mediterranean taxa in the Dead Sea region. The first scenario (Fig 11A) suggests a well-mixed balance of the three biome types with an increased probability for the Irano-Turanian biome compared to today. The MIS 3 environmental record represents an interplay of three biomes–not quite different from today, but less distinct along the biome interfaces. In order to compare biome distribution and human occupation, we plotted the Early Ahmarian site distribution (Fig 1) with both, the modern scenario biome model (Fig 11A) and the preferred MIS 3 scenario biome model (Fig 11B).

As a result, the Early Ahmarian sub-units NEA and SEA appear to mirror the biome distribution: the Mediterranean biome covering the Levantine coast and parts of its hinterland provided the habitat of the NEA (Fig 11). Focusing on the MIS 3 scenario (Fig 11A) the Mediterranean biome constituted a mosaic vegetation with forest and shrub components, offering grazing and browsing opportunities for multiple ungulate species. Thus, prey for human hunters of the NEA was available throughout the year. To the south, the Saharo-Arabian biome with sparse grassland and shrub vegetation covered the Negev and Sinai, including the Mediterranean coast of the Sinai, constituting the principal habitat of the SEA (Fig 11A). Towards the eastern margins of the SEA, the Irano-Turanian biome with its perennial herbs and shrubs featured along the escarpment zone east of the Jordan Rift. The most easterly outliers of the Early Ahmarian province are to be found lined up along the limestone escarpment. More to the east, on the limestone plateau, the dominating Irano-Turanian biome widely lacked Early Ahmarian settlements, probably due to scarcity of surface water (see [32] for more detailed information).

### Local findings: Archaeology, geology, paleobotany

Multidisciplinary research in the Dead Sea / southern Jordan Rift province allows for the exact tracing of the same time window in three different local records and thus for elucidating cultural and natural contexts of the Upper Palaeolithic during Greenland Isotope Stage (GIS) 8, around 38 ka cal BP.

Firstly, excavations at the open-air site of Al-Ansab 1, Lower Wadi Sabra, Ma´an Province/Jordan, have produced a new settlement area, securely attributed to the Early Ahmarian techno-cultural unit along its lithic assemblage classic to this Early Upper Palaeolithic archaeological entity. Radiometric dates indicate the human occupation to have taken place at c. 38 ka BP.

Secondly, detailed geological sampling and geochemical analysis of the framing sediments produced a 20 m long Quaternary sequence of valley fillings, attesting to mid-MIS 3 low-energy deposition of interlacing aeolic, fluvial and colluvial, local sands under mostly calm climatic conditions. Interlacing cemented layers attested to occasionally active soil formation processes and to corresponding halts of the depositional processes. Local sedimentation, in the Lower Wadi Sabra, terminated between 38 ka cal BP (^14^C) and 32 ka BP (OSL) to be continued in the Upper Wadi Sabra by further 25 m of sand depositions from the second half of MIS 3 and from MIS 2, until c. 18 ka BP.

Thirdly, the time window of the Early Ahmarian occupation and of the connected sediment deposits have been pinpointed within the vegetational sequence of the Dead Sea 5017-1-A core (Fig 10). The MIS 3 section of the core displays a decrease of forested areas at the onset of MIS 3, in favor of *Artemisia* dominated steppe and grassland elements. Vegetational change shows low-amplitude cyclical variation, but to slow and not dramatic enough to have impacted faunal and human populations. The overall picture is characterized by open landscapes and long-term sustainability of plant communities, throughout MIS 3. The time window under particular consideration, GIS 8, does not display any relevant differences to the time slices before and after. Altogether, the GIS 8 time-window saw calm, stabile climatic conditions with open vegetation, and long-term positive sedimentation budgets in the valleys, providing good preservation of the Al-Ansab 1 archaeological stratum.

## Discussion

The archaeological findings summarised above allow for evaluating the role of the Wadi Sabra within the Early Ahmarian overall settlement system. Such contextualisation must, necessarily, rely on the state of our present knowledge about site distribution and adaptation to regional environments, of the Early Ahmarian techno-cultural unit, largely focused on the Southern Levant.

### Human presence at the eastern margin of the Early Ahmarian

Given the importance of the Levantine Upper Palaeolithic featuring as an interface between Africa and Western Eurasia at the time of the MIS 3 massive AMH migration, any scope of discussing the role of the Early Ahmarian techno-cultural unit is limited still by many methodological constraints [2, 4, 5, 7, 18]. Archaeological evidence is still hampered by the paucity of detected sites, differences in excavation and collection methods, heterogeneity of cave and open-air sites and by chronological uncertainty.

Concerning our prospections in wadi sediments, we are aware that many sites were found when erosion had exposed artefacts and find layers [21–24]. Even after more than a decade of field research with predictive modeling of find-bearing sediments included [49], our understanding of the Late Pleistocene human-climate interactions in Wadi Sabra remains incomplete. The advantage of having detected an Early Ahmarian occupation floor framed by sterile sediments allowed for detailed analysis of the MIS 3 wadi evolution, though always constrained by limitations in dating an admixture of eolian and fluvial/colluvial sands, partially not fully bleached, by the OSL method [30]. Contextualizing the Wadi Sabra cultural and depositional sequence, with the vegetation record available in the region, is inhibited by the broad spectrum of OSL dates achieved and must currently rely on the more consistent ^14^C dataset of the archaeological find layer.

**Fig 12:**
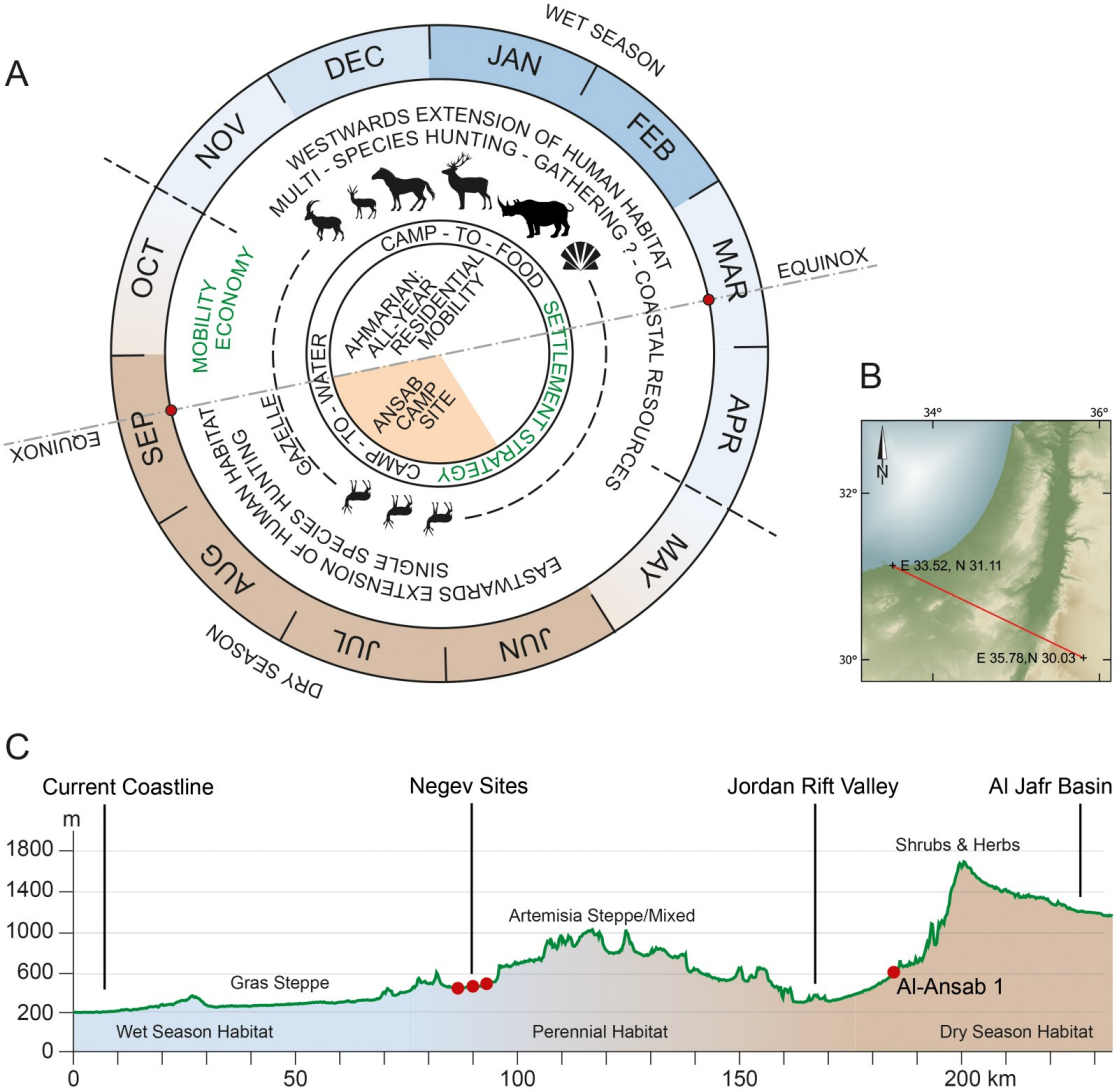
Hypothetic model of seasonal variability of the southern Early Ahmarian techno-cultural unit. (A) Annual cycle of habitat and economy with seasonal window of Al-Ansab 1 human occupation indicated. (B) West-East section through the annual habitat of the Southern Early Ahmarian techno-cultural unit. (Faunal data from Erg-el-Ahmar, Abu Noshra, Lagama, Boker: [64, Table 4.1]; Al-Ansab 1: CRC 806/H. Berke/N. Nolde (pers. Comm.); Precipitation (today): [71]). Coordinate System used: WGS 84/EPSG 4326.

Due to such constraints present also in the neighboring regions of Jordan (Wadi Al-Hasa, Jebel Qualkhah [4, 7, 59]) comparisons must be regarded as vague and preliminary, but still deserve attention as the only way to elucidate the role of the margins of the Early Ahmarian human habitat. A look to the regional neighborhood, east of Wadi Araba, shows that the archaeological observations made at Wadi Sabra do not feature as completely exceptional and isolated. The sequence of Upper Palaeolithic occupations and Quaternary valley sediments at Wadi Sabra (Fig 9A) [21–23, 30] compares to the Wadi Al-Hasa record, about 100 km to the North. Long-term archaeological surveys in the Wadi Al-Hasa have also produced a number of Upper Palaeolithic sites [60], including Early Ahmarian occurrences (e.g. the Tor Sadaf site; cf. Fig 1). Fundamentally reflecting local conditions, the MIS 3 sediment preservation in the Wadi Sabra and in Wadi Al-Hasa both allowed for almost the entire Upper Palaeolithic and Epipaleolithic sequence to be present. About 40 km to the south from Al-Ansab 1, an Early Ahmarian assemblage has been discovered at the Tor Fawaz site [59], in the Jebel Qualkha region. The mentioned Jordanian sites are, like Al-Ansab 1, examples of camp sites east of the Wadi Araba, at the easternmost margins of the Early Ahmarian range. Seashells excavated at Al-Ansab 1 and Tor Fawaz [59] attest to coastal relations and to westwards (Mediterranean coast) or southwards (Red Sea coast) connections (cf. [59, 61]) of the human occupants. Compared to the few Jordanian occurrences mentioned, the Early Ahmarian settlements to the far West of the Rift Valley represent more frequent and intensive presence of humans: some of the Negev and Sinai Early Ahmarian sites [4, 62–67] advocate for repeated presence of humans throughout the year, with a wide spectrum of hunting prey attested such as ibex, gazelle, horse, deer and even rhino [64, Table 4.1; 65]. This suggests the Jordanian sites as eastern components of a territory that had its centre further westwards, with site clusters existing in the Negev and in northern Sinai to be included in order to achieve a complete picture of the reconstructed regional range of annual mobility (Fig 12).

### Human mobility during the Early Ahmarian

According to ethnographic evidence on hunter-gatherer mobility patterns in arid environments (see [66] 93–104), wet season mobility is expected to depend on the best hunting opportunities with preference of camp sites close to the pathways of animals (labelled as „camp-to-food" strategy in Fig 12) and, by contrast, dry season mobility is expected to depend on reliable springs with camp sites chosen accordingly (labelled as „camp-to-water”strategy in Fig 12). We assume here that Al-Ansab 1, with its nearby spring, matches the second pattern. It has been previously argued that the bearers of the Early Ahmarian techno-cultural unit used to live in small family bands maintaining a circulating, residential mobility system [67] throughout the year, with short stays at camp sites and frequent changes of camps. As the camps were often related („tethered“) to nearby resources [62, 63, 68–70] such as game, water or raw materials this would indicate „tethered foraging”(see [66] 91–93), mostly seen in connection with „logistic”mobility ([66] 95). By contrast, our data do not support the notion of „logistic mobility”within the Early Ahmarian techno-cultural unit, with base camps, occupied for longer periods, supplied by expedition groups [66, 67]. Such logistic pattern must include special-task satellite sites, such as hunting locales or overnight camps scattered around the base camp. Among Early Ahmarian occurrences, however, separate special-task locales have not been found so far, neither within the camp sites, nor outside the residential camps. Both, intra-site comparisons (site plans, see below) and inter-assemblage comparisons (full list of Ahmarian lithic tool classes, see below and S2 Table) fail to prove neither any special-task areas within the campsites nor any special-task locales with lithic assemblages specialised on particular activities:

#### Comparison of intra-site patterns

Only the site plans available from Boker A [63], Abu Noshra I, Abu Noshra II [68] and Al-Ansab 1 (Fig 7) allow for direct insights into the placement of remnants discarded by human groups during their stay at the sites. All four site plans share common characteristics, displaying artefact clusters with all tool classes present in each cluster ([63] Fig 11.10 and [68] Fig 12.3 and 12.6). Generally, all stages of the reduction sequence were present in the assemblages, with no separate activity zones involved inside the occupation floor, suggesting that stone artefact production was not differentiated by spatial behavior [62, 63, 31, 69]. Fireplaces are in flat pits adjacent to the artefact scatters. Distinct areas indicating separately executed, special-task activities do not occur among the Early Ahmarian site plans, the artefact scatters consequently interpreted as products of repeated, multi-purpose and residential occupation events.

#### Interassemblage comparisons

**Fig 13:**
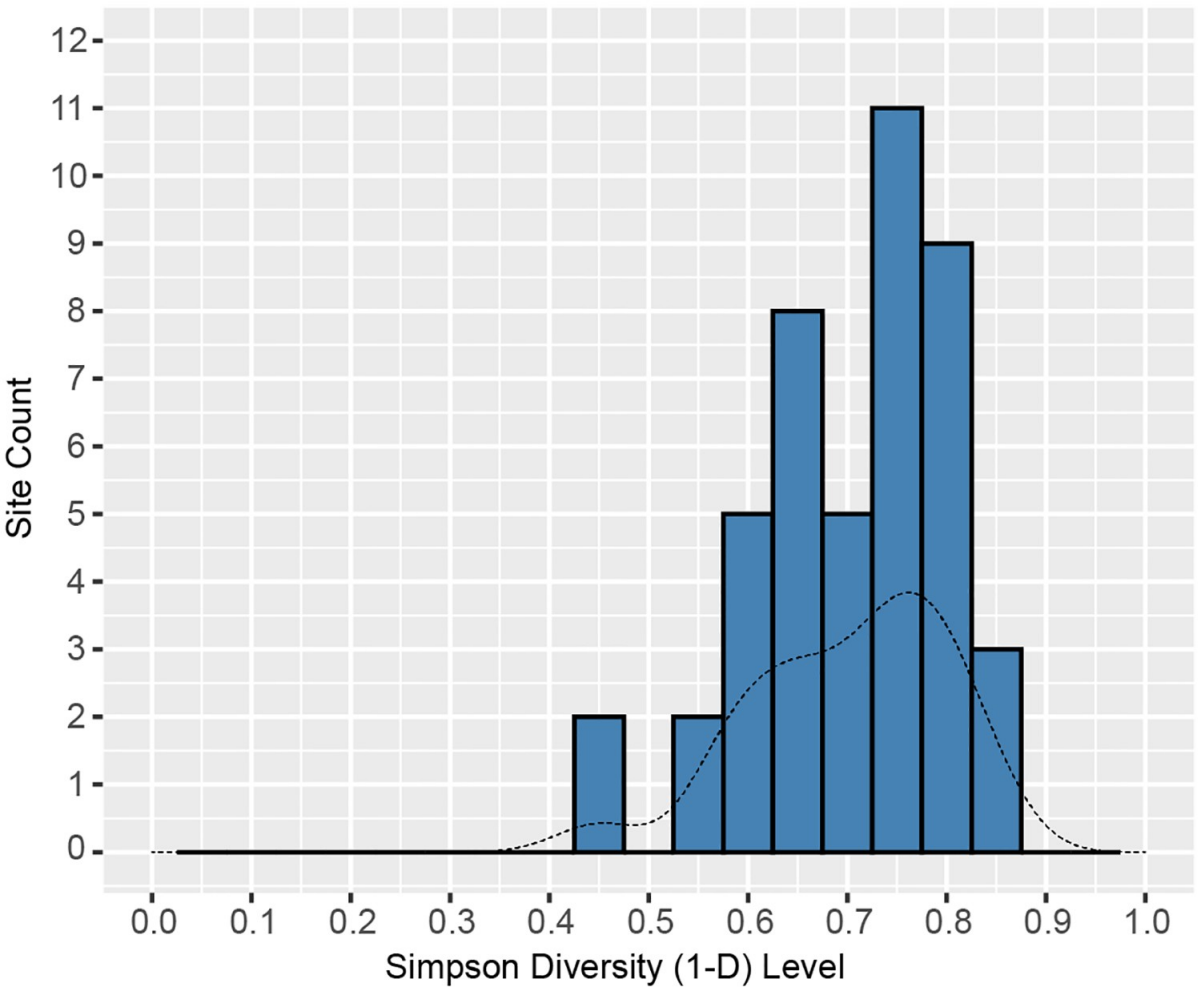
Histogram of site diversity and site-count of Early Ahmarian assemblages. The Simpson diversity (1-D) is represented by 0.1-increments on the x-axis while on the y-axis the absolute number of sites in the respective category is shown. A kernel-density line (dashed line) is also fitted to the diagram. For database see S2 Table.

To contextualize the Al-Ansab 1 lithic assemblage as either of special-task nature (specialized on certain tool classes, with low overall tool diversity) or as of multi-purpose nature (high tool diversity), we collected all tool counts available from published Early Ahmarian lithic assemblages (S2 Table). We calculated the Simpson diversity index for all tool classes occurring in the same assemblages, and we present here a comparative table of overall tool assemblage diversity (Fig 13). As a result, high tool assemblage diversity is attested for all known Early Ahmarian assemblages. Accordingly, each single assemblage mirrors the complete spectrum of activities that were regularly carried out by the bearers of the Early Ahmarian. This observation holds for both, large and small assemblages. Highly specialized (i.e. low diversity) assemblages are completely lacking. Even though taphonomic impact and limited site visibility of more specialized locations must always be considered, the aforementioned assemblage-based characteristics make for a strong point of generally high residential/circulating mobility and low degree of logistic complexity among foragers in this region and time, thus confirming the original model put forward by Marks and Friedel [67]. Though exceptional in its marginal location, the Al-Ansab 1 assemblage matches all mentioned characteristics of the Early Ahmarian group of sites, such as presence of all stages of reduction sequences and high diversity of tools.

Resulting from the above site and assemblage comparisons we propose a model of Early Ahmarian annual mobility—not as a final solution but rather to serve as a questionnaire for future research (Fig 12): we assume the Mediterranean sites to the North along with the Negev and Northern Sinai sites to indicate multi-seasonal human presence and the marginal sites in Transjordan (possibly along with the Southern Sinai sites) ephemeral, seasonal human presence, the attribution to the dry or wet season remaining rather assumptions than results. As one of the next research steps in refining or rejecting this model, future re-analysis of the lithic raw materials involved in each Early Ahmarian assemblage is needed to identify distant locales of connected mobility ranges.

### Regional patterns and trends

The millennia before the onset of the Early Ahmarian appear, at the present state of research, as a patchwork of anthropological and cultural facts not yet fully understood:

#### Anthropological background

The Jordan Rift Valley appears as one possible corridor of early AMH migration from Africa to Eurasia [see e.g. 18, 72–77] between 55–37 ka cal BP when AMH became permanently established in the region: The „Out-of-Africa IIb”AMH migration into the Middle East coincided with a major cultural turnover, labelled the “Middle-to-Upper Palaeolithic Transition” [78–81]. The final demise of the Neanderthals [82] and the sustainable establishment of AMH [83, 84 for earlier, possibly unsuccessful or deviated migrations and the role of the Arabian Peninsula see e.g. 85–94] occurred more or less simultaneously with the onset of the Initial Upper Palaeolithic [IUP; see e.g. 47, 92]: At Ksar Akil, the human fossil named “Ethelruda”, controversially debated as either belonging to the Neanderthals or to AMH [95, 96], was found along with the so-called “Initial Upper Palaeolithic” (IUP) techno-cultural unit, and an AMH fossil (named “Egbert”) was associated with the Early Ahmarian techno-cultural unit [97], which is the earliest fully Upper Palaeolithic culture found in the Ksar Akil sequence [41, 42, 98]. The chronological boundary between the IUP and the Ahmarian is thought to be around 45 ka BP. At Manot Cave, an AMH fossil has recently been dated to around 55 ka BP by the U/Th method [99]. The fossil lacks an associated cultural assemblage, but the obtained date coincides with the interface from the latest Middle Palaeolithic to the earliest IUP [9]. Consequently, both Neanderthals and AMH are presently candidates to have created the Initial Upper Palaeolithic, whereas the Early Ahmarian was exclusively tied to AMH [e.g. 12, 18]. As the Early Ahmarian postdates the Neanderthal/AMH anthropological transition by several millennia, this paper does not aim to compare the Ahmarian with Neanderthal adaptation (for recent, regional findings about Neanderthal land use see [100, 101]). We suggest that Neanderthals had already been extinct at the time of the Early Ahmarian, particularly its second half.

If early AMH really used the 56–44 ka BP humid interval to expand their habitat from Northeastern Africa into the Levant, as recently suggested from palaeobotanic evidence [16, see also 102], the immigrants had then arrived in a refuge offering relatively stable environmental conditions during the whole of MIS 3 [33]. Since the onset of MIS 3, the dramatic retreat of the northern Lebanese forests (Fig 10; Yammouneh record) allowed for the extension of steppe elements and for the integration of the Northern Levantine vegetation zone with its southern neighborhood. At the same time, steppe vegetation had already expanded into former desert areas of the southern Levant. Thus, a vegetational south-to-north corridor [33] had already opened up in the Levant, before the mentioned 56–44 ka BP humid interval supposedly came to additionally trigger AMH immigration. Consequently, the environmental change reported here had already begun several millennia before the occurrence of AMH. The last Neanderthals would have profited from the same extension of open landscapes just like the immigrating AMH which means that environmental change cannot alone explain the exchange from Neanderthal to AMH populations.

#### A northern and a southern division of the Early Ahmarian

After the humid interval, the following three thousand years (see S3 Table for ^14^C dates) in the Levant saw the emergence of a new cultural context (Early Ahmarian) exclusively connected with AMH. The mentioned (see introduction) NEA and SEA subgroups (Fig 1) refer to either production of broad blades from bipolar volumetric cores resulting in (partially-) backed blades and relatively massive El-Wad points, all classical to the NEA, or, in contrast, to unipolar volumetric blade and bladelet production promoting slender El-Wad points as the defining criteria for the SEA. Furthermore, quite a few sites in the Levant contain typical Early Ahmarian features along with (presumably much later) Levantine Aurignacian artefacts [13,14, 29, 101–105]. Though the Levantine Aurignacian is originally to be defined by flake production, carinated elements and Dufour bladelets, in some cases these characteristics occurred alongside El-Wad points and blades all classic to the Early Ahmarian. The north-south division is debatable, since there are claimed occurrences of the SEA as far north as northern Syria (e.g. Umm el Tlel and Wadi Kharar 16R; [5, 106]. Thus, the NEA/SEA division might reflect differences in economic behavior, such as intensification of raw material exploitation, either within one and the same social context or within different social entities adapted to different landscapes in the North and in the South. To evaluate these options, a closer look to the relevant timescale is needed:

#### The NEA and SEA during the first and second half of the Early Ahmarian

**Fig 14:**
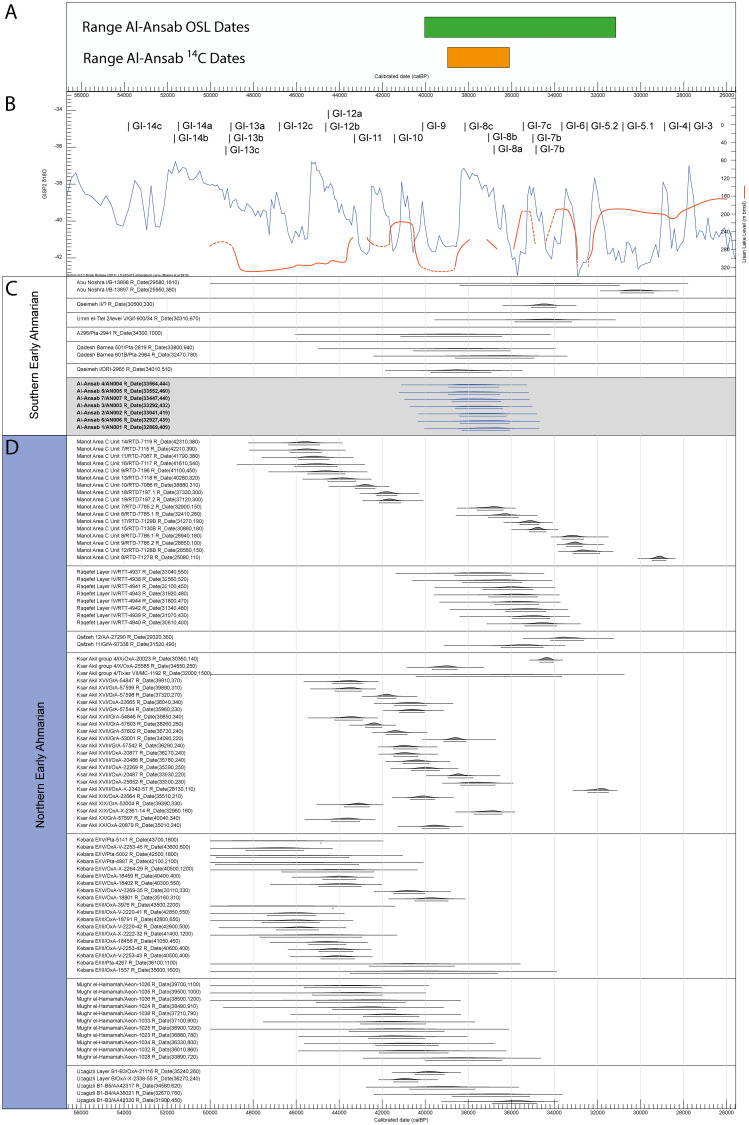
Chronology of the Early Ahmarian techno-cultural unit at local, regional and global scale. (A) Al-Ansab 1: time range (two-sigma) of pleistocene wadi fill according to combined OSL dates (green bar) and time range (two-sigma, cal BP) of combined 14C-dates of the Al-Ansab 1 settlement site (orange bar). (B) GISP2 δ^18^O Curve (blue) and Lisan Lake levels (orange) ranging from c. 57 ka BP until c. 26 ka BP. (C) Calibrated 14C dates for sites of the Southern Early Ahmarian. (D) Calibrated 14C dates for sites of the Northern Early Ahmarian. The human occupation of Al-Ansab 1 (A) matches, on a regional scale (C), the second half of the main occupation phase of SEA sites around 39–37 ka cal BP and, on a global scale (B), the onset of D/O cycle 18 with warming event GIS 8 [107] as the most probable global climatic context at the time of the Al-Ansab 1 occupation. 14C dates were calibrated with OxCal 4.3 using the IntCal 13 calibration record [108], see S3 Table for references. GISP 2 record according to [107; see also 109, 110].

Whereas the first half of the Early Ahmarian (mostly the NEA; Fig 14) included some dry and cool phases (culminating at the Heinrich Event HE 4 cooling of the northern hemisphere that was also detected in several Levantine records [e.g. 15, 57, 50, 51], the second half (38.5–36 ka BP; Fig 14) of the Early Ahmarian (both NEA and SEA) matches with a prominent humid and warm interval (Greenland Interstadial GIS 8) which terminated contemporaneously with most of the Early Ahmarian settlements around 36 ka BP (Fig 14). If the chronological offset of some three thousand years, between the onset of NEA and the onset of SEA, would hold (Fig 14), the Early Ahmarian might have originated in the northern Levant and then expanded (around 38.5 ka BP) into what became the southern province of the Early Ahmarian (SEA) comprising the Negev, the Sinai Peninsula and western Transjordan. The Al-Ansab 1 Early Ahmarian occupation consequently documents the height of the Early Ahmarian habitat expansion, as one of the most easterly outliers of the SEA featured under the most favorable and stable environmental conditions, as documented (see below) in the regional vegetation record.

### Vegetation and human occupation at Levantine scale

The archaeological site distribution of the Early Ahmarian and the connected NEA/SEA techno-cultural division, along with its observed congruence to biome patterns, rise the question when and how environmental conditions came to interplay with human occupation. To tackle this question we place the time range under consideration into the regional vegetation history and discuss explanations of the mentioned congruence.

#### MIS 3 vegetation history of the Levant

In order to set our results from Dead Sea core 5017-1-A [33, 34] into the regional context we compare data sets (Fig 10) from the 9095 marine core (Eastern Mediterranean) [50], Yammouneh (Lebanon) [51] and Tenaghi Philippon (Greece) [52, 53] that all show some similarities but also major differences. First of all, vegetation shifts at the northern Tenaghi Philippon location correspond closely with North Atlantic climate changes depicted for example in the North Greenland Ice Core Project (NGRIP) isotope record (Fig 10, see also [109, 110]). Rapid and strong vegetation changes in Greece correspond to stadial-interstadial cycles [52, 53]. In contrast, vegetation responses in the Levant were less extreme resulting in more stable conditions. Although Levantine environmental conditions are also influenced by the North Atlantic climate, the signal is buffered probably due to the distance and other climatic influences. Secondly, the Levantine records represent the formation of a steppe corridor along the entire Levant. In the mountainous areas of the northern Levant, woodland density drastically decreased after 60 ka BP leading to an open steppe vegetation until the end of the last glacial. This substantial deforestation is suggested by decreased arboreal pollen frequencies at Yammouneh. In addition, southern desert areas turned into steppe vegetation in the cause of the last glacial. *Artemisia* values were steadily increasing in the Dead Sea and core 9095 pollen records suggesting a gradual cooling and a spread of steppe. Biome modeling illustrates this vegetation change during MIS 3 and drafts the extent of the steppe corridor (Fig 11 [24, 33]). After 40 ka BP, pollen ratios of the Yammouneh record show a reduction of AP, *Artemisia* and Amaranthaceae percentages and an increase of taphonomically favoured Chicoriodeae. Though probably caused by local taphonomic processes, it still suggests ongoing dry and cold conditions in the northern Levant [51]. The southern Levantine pollen records suggest a further expansion of steppe and the coldest conditions during MIS 2 [33, 50].

To summarise, the period in question begun with a marked difference between desert and steppe vegetation in the south and more forested vegetation in the north. This situation prevailed until c. 60 ka BP, when arboreal vegetation decreased strongly at Yammouneh, steppe vegetation increased steadily in the southern parts and a tendency towards alignment between south and north can be observed. In the course of this process, open landscapes with steppe vegetation became the dominating biome in the region. This indicates a change of climate towards cooler conditions (with an average of -3° during MIS 3 below the present mean [33]; see also following section). In addition, the water availability for plants (effective moisture) decreased in the high altitude northern parts mainly due to orographic effects [111]. At the same time, the effective moisture increased in the southern parts probably due to lower evapotranspiration [33]. Therefore, sufficient moisture was available in the system for e.g. the described vegetation change and Lisan lake level rise without necessarily increasing the precipitation (see climate and lake level modeling [112]. Such calm climatic background might also explain the accumulation of huge deposits, at the same time, in Wadi Sabra: lowering winter temperatures, as attested at Lake Lisan, increased weathering intensity [113] of the sandstones surrounding Wadi Sabra, thus producing more debris adding to wadi fillings.

#### Three biomes and NEA/SEA human occupation

The biome model (Fig 11) displays the preference of the northern group of Early Ahmarian sites (NEA) for the Mediterranean biome, and the more diverse biome context of the southern group of sites (SEA), which would possibly have been connected with seasonal patterning of biomes exploited by human populations. The relationship of the NEA with the Mediterranean biome and the SEA with the Saharo-Arabian biome (as indicated in Fig 11B) underlines the grouping of the Early Ahmarian into NEA and SEA, respectively. Given the SEA limited to the second part of the Early Ahmarian chronology (Fig 14), the NEA distribution mirrors 8 ka of human adaptation to the Mediterranean biome (from GIS 12 to GIS 8), and the SEA distribution an expansion phase of 5 ka (GIS 9 to GIS 8) which resulted in peopling the Saharo-Arabian biome to the south.

In our view, the Mediterranean coast, the Negev and the Transjordan upland regions formed the three elements of an annual mobility range of prehistoric foragers, predominantly travelled in an East-West direction and vice versa during the year. The difference between woodlands in the north and steppe/desert in the south had decreased in favor of a growing component of *Artemisia* steppe. From the vegetational aspect, the *Artemisia* steppe dominating the Irano-Turanian biome to the east would even offer better conditions for ungulates, constituting the primary prey of Pleistocene hunters. On the karst plateau to the east, however, settlement sites are widely lacking because of water scarcity. This can explain marginal Early Ahmarian sites to be strictly limited to the escarpment zone lined up with many springs.

## Conclusions

The establishment of the Ahmarian techno-cultural unit, during the pronounced global warm interval at 45–43 ka BP (Greenland Interstadial GIS 12) and the maximum expansion of the Early Ahmarian sub-groups NEA and SEA during the equally pronounced global warm interval at 39–36 ka BP (Greenland Interstadial GIS 8) represent the AMH demographic correlate of the extension of an environmental corridor that had come into action since 60 ka BP. The emergence and successful dispersal of the Early Ahmarian culture and the connected permanent establishment of AMH were both based on the environmental integration of the Levantine corridor. AMH populations migrating into the Levant during MIS 3, all profited from the refugial capacity of the relatively stable Levantine natural environment constituted by the Saharo-Arabian biome, the Mediterranean biome and the Irano-Turanian biome.

Since ~60 ka BP, the latter of them offered one (in terms of vegetation) quite homogenous and long-lasting, large south-to-north corridor from southern Jordan to the northern Lebanon mountains. Around 45 ka BP, and inside the same corridor, the introduction or invention, of hunting weapons with lithic projectile inserts, marked the onset of the Early Ahmarian techno-cultural unit, exclusively tied to AMH populations. We assume the Ahmarian techno-cultural unit came into play within a regional framework, at mid-MIS 3, of slowly lowering annual mean temperature and slow decrease of precipitation. This change, which is mirrored in the local sedimentary archives at Wadi Sabra is also reflected by the supraregional re-organisation of vegetational patterning within the three biomes which constitute the Levantine landscapes. In particular, the vegetational difference between southern desert environments (southern Jordan) and northern woodlands (Lebanon) areas had diminished after 60 ka BP, and again around 40 ka BP, in favor of *Artemisia* and grass steppe covering large parts of the whole Levant.

We consider the mosaic landscapes of the Mediterranean biome to have constituted the original habitat of the Early Ahmarian including forest and steppe components. We hypothesize that, later on, the carriers of the Early Ahmarian increasingly focused on exploitation of steppe landscapes which led to expanding their habitat into the Saharo-Arabian biome. The undramatic, long-term change in favor of more open landscapes would explain the difference, in time and space, between the NEA and SEA sub-units of the Early Ahmarian. The NEA/SEA northern and southern division of the Early Ahmarian is founded in known differences of lithic technology, with narrow fronted cores and narrow projectile points mainly in the south (with 2 exceptions) and broader fronted cores and points in the north. The exact significance of this technological patterning remains still unclear, but besides the mentioned regional preferences, environmental change gains importance as one of its driving forces.

The early part of the NEA (45 to 43 ka BP), matching with the Mediterranean biome, represents the initial phase of the Early Ahmarian, which, from 43 ka BP, expanded into the Saharo-Arabian biome (SEA sub-unit). The later part of the NEA continued to exist in the Mediterranean biome, contemporaneously to the adjacent SEA occupation. The expansion phase culminated in the 39–36 ka BP global warming event GIS 8. SEA humans had now expanded into a steppe and semi-desert biome connected with more pronounced differences between the dry and the wet seasons. Seasonality consequently separated central, perennial habitats on the one hand (NEA: the northern Levant and SEA: Negev/Northern Sinai) from marginal, seasonal habitats on the other hand (SEA: southern coastal strip, southern Sinai and eastern Transjordan).

In contrast to its accepted role as a stepping stone within the south-to-north expansion of AMH on their long way from Africa to Eurasia, the Early Ahmarian techno-cultural unit displays, internally, a pronounced north-to-south tendency of expansion.

## Supporting information

S1 Table List of Early Ahmarian archaeological sites. The IDs in the list correspond to the IDs used in both the map (Fig 1) and the diversity evaluation in S2 Table.
(DOCX)

S2 Table Diversity of lithic tools in Early Ahmarian assemblages. (DOCX)

S3 Table ^14^C Dates of southern and northern Ahmarian assemblages. Drawn from the indicated literature and the present study as presented in Fig 14. IDs correspond to S1 and S2 Tables.
(DOCX)

S4 Table Detailed analysis of Al-Ansab 1 raw material units and their associated cores, debitage and tools. This table contains data from the material excavated in the campaigns 2009 until 2014.
(DOCX)

S5 Table Sources and characteristics of geodata used in this paper. (DOCX)

S6 Table Related databases of the CRC 806. (DOCX)
