# Supplementary material for: Al-Ansab and the Dead Sea: Mid-MIS 3 archaeology and environment of the early Ahmarian population of the Levantine corridor
Source: PLoS One. 2020 Oct 13;15(10):e0239968. doi: 10.1371/journal.pone.0239968 (PMC7553344; doi:10.1371/journal.pone.0239968)
Supplement: S1 Table — The IDs in the list correspond to the IDs used in both the map (Fig 1) and the diversity evaluation in S2 Table. (DOCX) [file pone.0239968.s001.docx]

| **ID** | **Name** | **Longitude** | **Latitude** | **NEA/SEA** |
| --- | --- | --- | --- | --- |
| 1 | Abu Noshra I | 33.94 | 28.694 | SEA |
| 2 | Abu Noshra II | 33.939 | 28.694 | SEA |
| 3 | Abu Noshra VI | 33.941 | 28.696 | SEA |
| 4 | Tor Fawaz | 35.568 | 29.474 | SEA |
| 5 | Wadi Sudr 6 | 33.068 | 29.784 | SEA |
| 6 | Tor Aeid (J432) | 35.338 | 29.937 | SEA |
| 7 | Tor Hamar (J431) | 35.319 | 29.939 | SEA |
| 8 | Jebel Humeima (J412) | 35.337 | 29.957 | SEA |
| 9 | Al -Ansab | 35.383 | 30.234 | SEA |
| 10 | Ein Qadis IV | 34.499 | 30.574 | SEA |
| 11 | Ein Qadis V | 34.492 | 30.574 | SEA |
| 12 | Ein Qadis III | 34.492 | 30.578 | SEA |
| 13 | Lagama XI | 33.433 | 30.633 | SEA |
| 14 | Qadesh Barnea 601, 602, 9 | 34.417 | 30.633 | SEA |
| 15 | Qadesh Barnea 501 | 34.402 | 30.642 | SEA |
| 16 | Qseimeh II | 34.391 | 30.654 | SEA |
| 17 | Qseimeh I | 34.39 | 30.657 | SEA |
| 18 | Qseimeh III | 34.38 | 30.666 | SEA |
| 19 | Lagama V | 33.438 | 30.766 | SEA |
| 20 | Lagama XVI | 33.439 | 30.766 | SEA |
| 21 | Lagama VI | 33.437 | 30.767 | SEA |
| 22 | Lagama VII | 33.439 | 30.767 | SEA |
| 23 | Lagama XV | 33.434 | 30.768 | SEA |
| 24 | Lagama VIII | 33.446 | 30.772 | SEA |
| 25 | Lagama XII | 33.452 | 30.776 | SEA |
| 26 | Ain al-Buhayra (WHS 618) | 35.928 | 30.839 | SEA |
| 27 | EHLPP 1 lower | 35.928 | 30.839 | SEA |
| 28 | Boker A | 34.776 | 30.84 | SEA |
| 29 | Boker D | 34.775 | 30.84 | SEA |
| 30 | Sde Divshon 27B | 34.765 | 30.84 | SEA |
| 31 | Tor Sadaf | 35.964 | 30.844 | SEA |
| 32 | WHS 623X | 35.921 | 30.848 | SEA |
| 33 | WHNBS 68 | 35.928 | 30.852 | SEA |
| 34 | Nahal Nizzana XIII | 34.44 | 30.876 | SEA |
| 35 | Multaqa al-Widyan (WHNBS 195) | 35.906 | 30.885 | SEA |
| 36 | El Quseir D | 35.328 | 31.578 | SEA |
| 37 | Erq el-Ahmar D-F | 35.324 | 29.936 | SEA |
| 38 | Mughr el-Hamamah | 35.634 | 32.257 | NEA |
| 39 | Raqefet | 35.051 | 32.391 | NEA |
| 40 | Kebara Unit IV | 34.937 | 32.559 | NEA |
| 41 | El-Wad | 34.967 | 32.667 | NEA |
| 42 | Qafzeh | 35.318 | 32.688 | NEA |
| 43 | Manot cave | 35.193 | 33.035 | NEA |
| 44 | Antelias | 35.6 | 33.91 | NEA |
| 45 | Ksar Akil | 35.61 | 33.911 | NEA |
| 46 | Yabroud II | 36.645 | 33.977 | NEA |
| 47 | Koubbah II | 35.659 | 34.272 | NEA |
| 48 | Abou Halka IVd | 35.808 | 34.403 | NEA |
| 49 | Umm el Tlel 2 V | 38.897 | 35.262 | SEA |
| 50 | Umm el Tlel 2 XI | 38.897 | 35.262 | SEA |
| 51 | Ücagizli | 35.977 | 36.004 | NEA |
| 52 | Kanal cave | 35.931 | 36.107 | NEA |
