# Supplementary material for: Al-Ansab and the Dead Sea: Mid-MIS 3 archaeology and environment of the early Ahmarian population of the Levantine corridor
Source: PLoS One. 2020 Oct 13;15(10):e0239968. doi: 10.1371/journal.pone.0239968 (PMC7553344; doi:10.1371/journal.pone.0239968)
Supplement: S2 Table — (DOCX) [file pone.0239968.s002.docx]

| **ID** | **SEA/NEA** | **Name** | **Endscraper** | **Burin** | **Perforatir** | **Truncation** | **Denticulate** | **Edge retouch** | **El-Wad point** | **Backed/Finely retouched** | **Various** | **Total** | **Simpson Diversity (1-D)** | **Literature** |
| --- | --- | --- | --- | --- | --- | --- | --- | --- | --- | --- | --- | --- | --- | --- |
| 1 | SEA | Abu Noshra I | 0 | 26 | 10 | 20 | 8 | 25 | 0 | 73 | 3 | 165 | 0.74 | Saca 2002 |
| 2 | SEA | Abu Noshra II | 21 | 39 | 9 | 5 | 25 | 98 | 0 | 170 | 26 | 432 | 0.73 | Saca 2002 |
| 3 | SEA | Abu Noshra VI | 1 | 4 | 1 | 4 | 0 | 6 | 0 | 20 | 0 | 36 | 0.64 | Saca 2002 |
| 4 | SEA | Tor Fawaz 1980s & 1994 | 7 | 6 | 1 | 10 | 14 | 6 | 1 | 6 | 5 | 102 | 0.85 | Kerry and Henry 2003 |
| 5 | SEA | Wadi Sudr WS6 | 23 | 5 | 0 | 0 | 4 | 1 | 0 | 55 | 9 | 97 | 0.61 | Baruch and Bar-Yosef 1986 |
| 6 | SEA | Tor Aeid | 18 | 18 | 3 | 3 | 8 | 3 | 9 | 14 | 6 | 82 | 0.84 | Saca 2002; Coinman and Henry 1995 |
| 7 | SEA | Tor Hamar | 11 | 19 | 1 | 4 | 1 | 12 | 7 | 2 | 3 | 83 | 0.8 | Saca 2002; Coinman and Henry 1995 |
| 8 | SEA | Jebel Humeima | 10 | 6 | 3 | 5 | 20 | 4 | 0 | 32 | 3 | 114 | 0.77 | Saca 2002; Coinman and Henry 1995 |
| 9 | SEA | Al-Ansab 1 | 5 | 8 | 0 | 10 | 1 | 3 | 1 | 0 | 0 | 61 | 0.74 | Schyle 2015 |
| 10 | SEA | Ein Qadis IV | 52 | 13 | 2 | 6 | 33 | 87 | 27 | 11 | 12 | 332 | 0.79 | Goring-Morris 1995; Saca 2002 |
| 13 | SEA | Lagama XI | 10 | 8 | 0 | 1 | 6 | 4 | 6 | 6 | 7 | 105 | 0.85 | Belfer and Bar-Yosef 1977 |
| 14 | SEA | Qadesh Barnea 9 | 27 | 31 | 4 | 40 | 127 | 353 | 26 | 101 | 8 | 718 | 0.7 | Saca 2002; Gilead and Bar-Yosef 1993 |
| 15 | SEA | Qadesh Barnea 501 | 2 | 3 | 0 | 0 | 4 | 0 | 0 | 12 | 1 | 22 | 0.64 | Gilead and Bar-Yosef 1993 |
| 16 | SEA | Quseimeh II | 2 | 1 | 1 | 1 | 2 | 5 | 0 | 2 | 0 | 30 | 0.8 | Saca 2002 |
| 17 | SEA | Quseimeh I | 37 | 2 | 0 | 2 | 16 | 21 | 3 | 3 | 2 | 93 | 0.72 | Saca 2002 |
| 18 | SEA | Quseimeh III | 30 | 18 | 0 | 4 | 3 | 54 | 5 | 44 | 6 | 164 | 0.77 | Saca 2002 |
| 19 | SEA | Lagama V | 12 | 14 | 0 | 1 | 9 | 11 | 26 | 220 | 4 | 326 | 0.44 | Belfer and Bar-Yosef 1977 |
| 20 | SEA | Lagama XVI | 1 | 3 | 0 | 0 | 12 | 3 | 22 | 51 | 2 | 103 | 0.63 | Belfer and Bar-Yosef 1977 |
| 21 | SEA | Lagama VI | 0 | 0 | 0 | 0 | 3 | 3 | 6 | 4 | 0 | 16 | 0.73 | Belfer and Bar-Yosef 1977 |
| 22 | SEA | Lagama VII | 2 | 23 | 0 | 6 | 13 | 14 | 423 | 419 | 4 | 903 | 0.57 | Belfer and Bar-Yosef 1977 |
| 23 | SEA | Lagama XV | 1 | 7 | 0 | 5 | 11 | 4 | 99 | 165 | 2 | 294 | 0.57 | Belfer and Bar-Yosef 1977 |
| 24 | SEA | Lagama VIII | 3 | 4 | 0 | 0 | 1 | 0 | 4 | 28 | 5 | 49 | 0.58 | Belfer and Bar-Yosef 1977 |
| 25 | SEA | Lagama XII | 7 | 15 | 3 | 1 | 17 | 7 | 2 | 85 | 2 | 152 | 0.59 | Belfer and Bar-Yosef 1977 |
| 28 | SEA | Boker A | 10 | 62 | 0 | 8 | 47 | 101 | 49 | 103 | 12 | 392 | 0.81 | Saca 2002 |
| 30 | SEA | Sde Divshon (27B) | 90 | 60 | 0 | 12 | 4 | 46 | 11 | 43 | 9 | 303 | 0.79 | Williams 2003 |
| 31 | SEA | Tor Sadaf | 45 | 9 | 0 | 0 | 0 | 40 | 150 | 83 | 8 | 336 | 0.7 | Fox 2003 |
| 34 | SEA | Nahal Nazzana XIII | 5 | 1 | 1 | 1 | 3 | 13 | 0 | 0 | 1 | 40 | 0.67 | Saca 2002 |
| 36 | SEA | El Quseir Level D | 36 | 6 | 0 | 0 | 4 | 6 | 0 | 25 | 2 | 78 | 0.68 | Perrot 1955 |
| 37 | SEA | Erq-el-Ahmar | 14 | 9 | 2 | 9 | 18 | 61 | 8 | 11 | 0 | 130 | 0.74 | Saca 2002 |
| 39 | NEA | Raqefet Layer IV | 15 | 4 | 1 | 4 | 9 | 12 | 0 | 7 | 3 | 55 | 0.82 | Lengyel 2007 |
| 40 | NEA | Kebara E/III | 181 | 13 | 1 | 3 | 7 | 34 | 43 | 7 | 7 | 296 | 0.59 | Saca 2002; Tostevin 2012 |
| 41 | NEA | El-Wad E | 1432 | 519 | 0 | 0 | 34 | 0 | 177 | 22 | 211 | 2394 | 0.58 | Saca 2002 |
| 42 | NEA | Qafzeh Layer E | 88 | 23 | 0 | 20 | 17 | 53 | 104 | 4 | 11 | 319 | 0.78 | Bar-Yosef and Belfer-Cohen 2004 |
| 45 | NEA | Ksar Akil XX sqs. E4 and F4 | 32 | 6 | 1 | 45 | 34 | 2 | 0 | 13 | 29 | 575 | 0.8 | Azoury 1986 |
| 46 | NEA | Yabroud II Layer 5 | 101 | 85 | 0 | 0 | 0 | 0 | 63 | 116 | 106 | 471 | 0.79 | Pastoors et al 2008; Ziffer 1981 |
| 50 | SEA | Umm el Tlel 2/14c | 1 | 3 | 0 | 0 | 1 | 1 | 0 | 7 | 0 | 13 | 0.64 | Ploux and Soriano 2003 |
| 51 | NEA | Ucagizli B1-B2 | 449 | 26 | 6 | 31 | 46 | 35 | 0 | 40 | 207 | 1150 | 0.64 | Kuhn et al. 2009 |
| 52 | NEA | Kanal | 172 | 4 | 0 | 15 | 5 | 0 | 33 | 51 | 57 | 370 | 0.68 | Kuhn et al. 1999 |

**Comments on supplementary file S2**

*Diversity calculations*

To assess the toolkit diversity of Early Ahmarian sites from the Eastern Mediterranean, legacy data on the typological composition of 45 lithic assemblages assigned to the Southern and Northern Ahmarian respectively have been collected from the literature. These data were first normalised to ensure their general comparability, i.e. sub-categories were merged into higher-order types whenever required. It was also ensured that categories which are rarely encountered and include only a few specimens were eliminated and their information merged with the ‘various’ data. In a second step, the lithic data was subjected to standard diversity analysis, using the statistical environment of PAST2 (Hammer et al. 2001), computing the Simpson index (1-D) for all recorded Ahmarian assemblages.

*Results and interpretation*

The diversity calculations demonstrate that the recorded Early Ahmarian assemblages yield highly diverse lithic toolkits with specialized assemblages widely lacking. There are no notable geographic or chronological trends in these values and both Simpson indices produce mean values above 0.6 [Northern Ahmarian mean = 0.7074; Southern Ahmarian mean = 0.6884]. In addition, there are no significant differences between the diversity measures derived for the Northern and Southern Ahmarian sites [p(same) = 0.99319]. The dispersion of the obtained Simpson values in statistical space is shown in **S2/fig. 1.** In total, the results demonstrate the Early Ahmarian yield relatively diverse tool kits. The overall pattern is interpreted as lending support to the hypothesis that the Early Ahmarian is the product of residential mobility (see Binford 1980; Kelly 1992), yet within systems of relatively short-term occupations (cf. Monigal 2003; Hussain 2013; Parow-Souchon 2016).
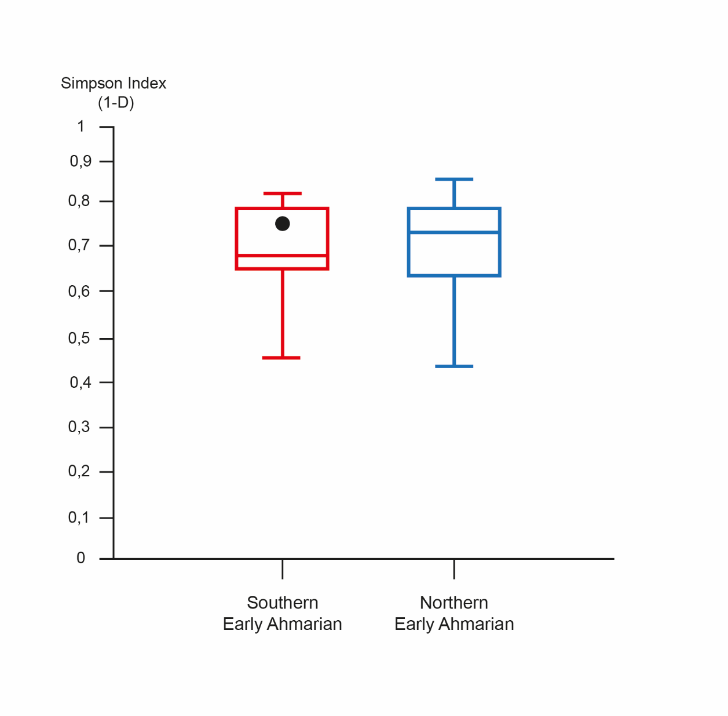


**S2/Fig. 1.** Boxplots of Simpson diversity indices for Southern (red) and Northern (blue) Early Ahmarian sites. Black dot represents the value obtained for Al-Ansab 1.

**S2/References**

Azoury I., Ksar Akil: Lebanon: Technological and typological analysis of the transitional and early Upper Palaeolithic Levels of Ksar Akil and Abu Halka. Vol. 1: Levels XXV-XII. Parts I-II. BAR Int. Ser. 289. Oxford: BAR Publishing; 1986.

Bar-Yosef, O., Belfer-Cohen, A., The Qafzeh Upper Paleolithic assemblages: 70 years later. Eurasian Prehistory 2004; 2: 145–180.

Baruch, U., Bar-Yosef, O., Upper Paleolithic Assemblages from Wadi Sudr, Western Sinai. Paléorient 1986; 12(2): 69-84.

Belfer, A., Bar-Yosef, O., The Lagaman Industry. In: O. Bar-Yosef, J.L. Phillips (eds.), Prehistoric Investigations in Gebel Maghara, Northern Sinai. Jerusalem: Hebrew University Press; 1977. pp. 42-84.

Binford, L.R., Willow Smoke and Dogs’ Tails: Hunter-Gatherer Settlement Systems and Archaeological Site Formation. American Antiquity 1980; 45(1): 4-20.

Coinman, N.R., Henry, D.O., The Upper Paleolithic Sites. In: D.O. Henry (ed.), Prehistoric Cultural Ecology and Evolution: Insights from Southern Jordan. New York/London: Plenum Press; 1995. pp. 133-214.

Fox, J.R., The Tor Sadaf Lithic Assemblages: A Technological Study of the Early Upper Palaeolithic in the Wadi al-Hasa. In: A.N. Goring-Morris, A. Belfer-Cohen (eds.), More than Meets the Eye. Studies on Upper Palaeolithic diversity in the Near East. Oxford: Oxbow Books; 2003. pp. 80-94.

Gilead, I., Bar-Yosef, O., Early Upper Paleolithic Sites in the Qadesh Barnea Area, NE Sinai. Journal of Field Archaeology 1993; 20: 265-280.

Goring-Morris, A.N.. Upper Paleolithic occupation of the Ein Qadis area on the Si-nai/Negev Border. Atiqot 1995; 27: 1-14.

Hammer, O., Harper, D.A.T., Ryan, P.D., PAST: Paleontological statistics software package for education and data analysis. Palaeontologia Electronica 2001; 4 (1): http://palaeo-electronica.org/2001_1/past/issue1_01.htm.

Hussain, S.T., Zum Problem leptolithischer Klassifikationseinheiten im Jungpaläolithikum. Eine Kritik des technotypologischen Zusammenhangs von Frühem und Spätem Ahmarien in Südjordanien anhand der Steinartefaktinventare von al-Ansab 1 und Taibeh 3. Unpublished Master Thesis, University of Cologne, 2013.

Kelly, R.L., Mobility/Sedentism: Concepts, Archaeological Measures, and Effects. Annual Review of Anthropology 1992; 21: 43-66.

Kerry, K., Henry, D.O., Tow Fawaz (J 403): An Upper Paleolithic Occupation in the Gebel Qalkha Area. In: A.N. Goring-Morris, A. Belfer-Cohen (eds.), More than Meets the Eye. Studies on Upper Palaeolithic diversity in the Near East. Oxford: Oxbow Books; 2003. pp. 171-184.

Kuhn, S.L., Stiner, M.C., Güleç, E., Initial Upper Palaeolithic in south-central Turkey and its regional context: a preliminary report. Antiquity 1999; 73: 505-517.

Kuhn, S.L., Stiner, M.C., Güleç, E., Ozer, I., Yılmaz, H., Baykara, I. (et al.), The early Upper Paleolithic occupations at Űçağızlı Cave (Hatay, Turkey). J Hum Evol. 2009; 56: 87–113.

Lengyel, G.. Upper Palaeolithic and Epipalaeolithic Lithic Technologies at Raqefet Cave, Mount Carmel East, Israel. British Archaeological Reports International Series 1681. Oxford: Archaeopress; 2007.

Monigal, K. 2003. Technology, Economy and Mobility at the Beginning of the Levantine Upper Paleolithic. In: Goring-Morris, A. N., Belfer-Cohen, A. (eds.), *More than Meets the Eye. Studies on Upper Palaeolithic Diversity in the Near East.* Oxford: Oxbow Books, 118-133.

Ohnuma, K., Ksar Akil, Lebanon: A technological study of the earlier Upper Palaeolithic levels of Ksar Akil. Vol. 3: Levels XXV-XIV. BAR Int. Ser. 426 (Oxford 1988).

Parow-Souchon, H. 2016. *The Wadi Sabra. A contextual approach to the Palaeolithic landscape*. Doctoral Dissertation, University of Cologne.

Pastoors, A., G.-C. Weniger, J.F. Kegler 2008. The Middle – Upper Palaeolithic Transition at Yabroud II (Syria). A Re-evaluation of the Lithic Material from the Rust Excavation. Paléorient 34: 47-65.

Perrot, J. 1955. Le Paléolithique supérieur d’El Quseir et de Masaraq an Na’aj (Palestine). Inventaire de la collection René Neuville I et II. Bulletin de la Société préhistorique de France 52(8): 493-506.

Ploux, S., Soriano, S., Umm el Tlel, une séquence du Paléolithique supérieur en Syrie centrale. Industries lithiques et chronologie culturelle. Paléorient 2003; 29, 5-34.

Saca, I.N., Reconfiguring the early Upper Paleolithic of the southern Levant: The bigger picture (unpubl. PhD Thesis, University of Illinois at Chicago (Chicago 2002).

Schyle, D.,The Ahmarian site of al-Ansab 1. In: Schyle, D., Richter, J. (eds.), Pleistocene Archaeology of the Petra Area in Jordan. Studien zur Prähistorischen Archäologie 5 (Rahden/Westfalen 2015), 91-130.

Tostevin, G., Seeing lithics: A middle-range theory for testing for cultural transmission in the Pleistocene (Oxford 2012).

Williams, J.K., Examining the boundaries of the Levantine Aurignacian. PhD Dissertation, Southern Methodist University, Dallas (Dallas 2003).

Ziffer, D. 1981. Yabrud Shelter II - A re-consideration of its cultural composition and of its relevance to the Upper-Paleolithic cultural sequence in the Levant. Quartär 31/32: 69-94.

Shea, J.J. 2013. *Stone Tools in the Paleolithic and Neolithic Near East: A Guide. Cambridge*: Cambridge University Press. pp. 117-160.
