# Supplementary material for: Al-Ansab and the Dead Sea: Mid-MIS 3 archaeology and environment of the early Ahmarian population of the Levantine corridor
Source: PLoS One. 2020 Oct 13;15(10):e0239968. doi: 10.1371/journal.pone.0239968 (PMC7553344; doi:10.1371/journal.pone.0239968)
Supplement: S3 Table — Drawn from the indicated literature and the present study as presented in Fig 14. IDs correspond to S1 and S2 Tables. (DOCX) [file pone.0239968.s003.docx]

| ID | **Site** | **Ahmarian affiliation** | **Sample ID** | **Material** | **Dating Method** | **UNCAL AGES** | **1 Sigma** | **CAL AGES** | **1Sigma** | **Source** |
| --- | --- | --- | --- | --- | --- | --- | --- | --- | --- | --- |
| 51 | Ucagizli B1-B3 | Northern Ahmarian | AA42320 | charcoal | ABA-AMS | 31900 | 450 | 35776 | 469 | Kuhn et al. 2009 |
|  |  | Northern Ahmarian | AA38021 | charcoal | ABA-AMS | 32670 | 760 | 36879 | 965 | Kuhn et al. 2009 |
|  |  | Northern Ahmarian | AA42317 | charcoal | ABA-AMS | 34580 | 620 | 39107 | 677 | Kuhn et al. 2009 |
| 51 | Üçağızlı Layer B | Northern Ahmarian | OxA-X-2338-55 | shell | AMS | 36270 | 240 | 40902 | 295 | Douka 2013 |
| 51 | Üçağızlı Layer B1-B3 | Northern Ahmarian | OxA-21116 | shell | AMS | 35240 | 260 | 39790 | 332 | Douka 2013 |
| 38 | Mughr el-Hamamah | Northern Ahmarian? | Aeon-1023 | charcoal | ABOx | 36880 | 780 | 41253 | 677 | Stutz et al. 2015 |
|  |  | Northern Ahmarian? | Aeon-1024 | charcoal | ABOx | 38490 | 910 | 42573 | 641 | Stutz et al. 2015 |
|  |  | Northern Ahmarian? | Aeon-1025 | charcoal | ABOx | 36900 | 1200 | 41115 | 1036 | Stutz et al. 2015 |
|  |  | Northern Ahmarian? | Aeon-1026 | charcoal | ABOx | 39700 | 1100 | 43549 | 852 | Stutz et al. 2015 |
|  |  | Northern Ahmarian? | Aeon-1028 | charcoal | ABA-AMS | 33890 | 720 | 38117 | 949 | Stutz et al. 2015 |
|  |  | Northern Ahmarian? | Aeon-1032 | charcoal | ABOx | 36010 | 860 | 40484 | 850 | Stutz et al. 2015 |
|  |  | Northern Ahmarian? | Aeon-1033 | charcoal | ABOx | 37100 | 900 | 41391 | 754 | Stutz et al. 2015 |
|  |  | Northern Ahmarian? | Aeon-1034 | charcoal | ABOx | 36330 | 800 | 40786 | 756 | Stutz et al. 2015 |
|  |  | Northern Ahmarian? | Aeon-1035 | charcoal | ABOx | 39500 | 1000 | 43393 | 776 | Stutz et al. 2015 |
|  |  | Northern Ahmarian? | Aeon-1038 | humid acids | ? | 37210 | 790 | 41527 | 642 | Stutz et al. 2015 |
|  |  | Northern Ahmarian? | Aeon-1036 | charcoal | ABOx | 38500 | 1200 | 42592 | 917 | Stutz et al. 2015 |
| 40 | Kebara E/IV | Northern Ahmarian | Pta-5141 | charcoal | ABA | 43700 | 1800 | 46088 | 1728 | Bar-Yosef et al. 1996 |
|  |  | Northern Ahmarian | Pta-5002 | charcoal | ABA | 42500 | 1800 | 45795 | 1965 | Bar-Yosef et al. 1996 |
|  |  | Northern Ahmarian | Pta-4987 | charcoal | ABA | 42100 | 2100 | 44139 | 963 | Bar-Yosef et al. 1996 |
|  |  | Northern Ahmarian | OxA-X-2264-29 | charcoal | ABOx | 40500 | 1200 | 40737 | 377 | Rebollo et al. 2011 |
|  |  | Northern Ahmarian | OxA-V-2269-35 | charcoal | ABA-AMS | 36110 | 330 | 39693 | 388 | Rebollo et al. 2011 |
|  |  | Northern Ahmarian | OxA-18801 | charcoal | ABOx | 35160 | 310 | 43900 | 499 | Rebollo et al. 2011 |
|  |  | Northern Ahmarian | OxA-18402 | charcoal | ABOx | 40300 | 550 | 46859 | 663 | Rebollo et al. 2011 |
|  |  | Northern Ahmarian | OxA-V-2253-45 | charcoal | ABA-AMS | 43600 | 600 | 43963 | 401 | Rebollo et al. 2011 |
|  |  | Northern Ahmarian | OxA-18459 | charcoal | ABOx | 40400 | 400 | 43963 | 401 | Rebollo et al. 2011 |
| 40 | Kebara E/III | Northern Ahmarian | OxA-X-2222-32 | charcoal | ABOx | 41400 | 1200 | 46104 | 522 | Rebollo et al. 2011 |
|  |  | Northern Ahmarian | OxA-V-2220-41 | charcoal | ABA-AMS | 42850 | 550 | 46085 | 612 | Rebollo et al. 2011 |
|  |  | Northern Ahmarian | OxA-18791 | charcoal | ABOx | 42800 | 650 | 45860 | 455 | Rebollo et al. 2011 |
|  |  | Northern Ahmarian | OxA-V-2220-42 | charcoal | ABA-AMS | 42600 | 500 | 44529 | 428 | Rebollo et al. 2011 |
|  |  | Northern Ahmarian | OxA-18458 | charcoal | ABOXx | 41050 | 450 | 44129 | 407 | Rebollo et al. 2011 |
|  |  | Northern Ahmarian | OxA-V-2253-42 | charcoal | ABA-AMS | 40600 | 400 | 44045 | 405 | Rebollo et al. 2011 |
|  |  | Northern Ahmarian | OxA-V-2253-43 | charcoal | ABA-AMS | 40500 | 400 | 46942 | 250 | Rebollo et al. 2011 |
|  |  | Northern Ahmarian | OxA-3976 | charcoal | ABA-AMS | 43500 | 2200 | 45738 | 164 | Housley 1994 |
|  |  | Northern Ahmarian | Pta-4267 | charcoal | ABA | 36100 | 1100 | 39766 | 1620 | Bar-Yosef et al. 1996 |
|  |  | Northern Ahmarian | OxA-1567 | charcoal | ABA-AMS | 35600 | 1600 | 39766 | 1620 | Hedges et al. 1992 |
| 45 | Ksar Akil XX | Northern Ahmarian | OxA-20879 | marine shell | AMS | 35010 | 240 | 39529 | 322 | Douka et al. 2013 |
| 45 | Ksar Akil XIX | Northern Ahmarian | OxA-X-2361-14 | marine shell | AMS | 32960 | 160 | 37045 | 362 | Douka et al. 2013 |
|  |  | Northern Ahmarian | OxA-22664 | marine shell | AMS | 35510 | 210 | 40108 | 273 | Douka et al. 2013 |
| 45 | Ksar Akil XVIII | Northern Ahmarian | OxA-20486 | marine shell | AMS | 35780 | 240 | 40415 | 312 | Douka et al. 2013 |
|  |  | Northern Ahmarian | OxA-25652 | marine shell | AMS | 33300 | 230 | 37548 | 474 | Douka et al. 2013 |
|  |  | Northern Ahmarian | OxA-20487 | marine shell | AMS | 33930 | 220 | 38393 | 259 | Douka et al. 2013 |
|  |  | Northern Ahmarian | OxA-22269 | marine shell | AMS | 35390 | 250 | 39969 | 313 | Douka et al. 2013 |
|  |  | Northern Ahmarian | OxA-20877 | marine shell | AMS | 36270 | 240 | 40902 | 295 | Douka et al. 2013 |
|  |  | Northern Ahmarian | OxA-X-2342-57 | marine shell | AMS | 28130 | 110 | 31938 | 262 | Douka et al. 2013 |
| 45 | Ksar Akil XVI | Northern Ahmarian | OxA-22665 | marine shell | AMS | 36040 | 340 | 40669 | 389 | Douka et al. 2013 |
| 45 | Ksar Akil group 4/Tixier VII | Northern Ahmarian? | MC-1192 | charcoal | ABA | 32000 | 1500 | 36306 | 1639 | Mellars and Tixier 1989 |
| 45 | Ksar Akil group 4/X | Northern Ahmarian? | OxA-25585 | marine shell | AMS | 34550 | 250 | 39075 | 305 | Douka et al. 2013 |
| 45 | Ksar Akil group 4/IX | Northern Ahmarian? | OxA-20023 | marine shell | AMS | 30360 | 140 | 34357 | 159 | Douka et al. 2013 |
| 42 | Qafzeh 11 | Northern Ahmarian | GifA-97338 | charcoal | ABA-AMS | 31520 | 490 | 35442 | 487 | Bar-Yosef and Belfer-Cohen 2004 |
|  |  | Northern Ahmarian | AA-27290 | charcoal | ABA-AMS | 29320 | 360 | 33411 | 375 | Bar-Yosef and Belfer-Cohen 2004 |
| 39 | Raqefet Layer IV | Northern Ahmarian | RTT-4940 | charcoal | ABA-AMS | 30610 | 400 | 34574 | 352 | Lengyel et al. 2006 |
|  |  | Northern Ahmarian | RTT-4942 | charcoal | ABA-AMS | 31340 | 480 | 35283 | 470 | Lengyel et al. 2006 |
|  |  | Northern Ahmarian | RTT-4944 | charcoal | ABA-AMS | 31800 | 470 | 35684 | 482 | Lengyel et al. 2006 |
|  |  | Northern Ahmarian | RTT-4941 | charcoal | ABA-AMS | 32100 | 450 | 36009 | 496 | Lengyel et al. 2006 |
|  |  | Northern Ahmarian | RTT-4939 | charcoal | ABA-AMS | 31070 | 430 | 35026 | 417 | Lengyel et al. 2006 |
|  |  | Northern Ahmarian | RTT-4938 | charcoal | ABA-AMS | 32560 | 520 | 36724 | 723 | Lengyel et al. 2006 |
|  |  | Northern Ahmarian | RTT-4943 | charcoal | ABA-AMS | 31920 | 480 | 35806 | 503 | Lengyel et al. 2006 |
|  |  | Northern Ahmarian | RTT-4937 | charcoal | ABA-AMS | 33040 | 550 | 37260 | 745 | Lengyel et al. 2006 |
| 45 | Ksar Akil XVI | Northern Ahmarian | GrA-54847 | shell | AMS | 39910 | 370/320 | 43581 | 360 | Bosch et al. 2015 |
|  |  | Northern Ahmarian | GrA-57544 | shell | AMS | 35960 | 230/210 | 40605 | 302 | Bosch et al. 2015 |
|  |  | Northern Ahmarian | GrA-57598 | shell | AMS | 37320 | 270/240 | 41784 | 210 | Bosch et al. 2015 |
|  |  | Northern Ahmarian | GrA-57599 | shell | AMS | 39890 | 310/280 | 43552 | 317 | Bosch et al. 2015 |
| 45 | Ksar Akil XVII | Northern Ahmarian | GrA-53001 | shell | AMS | 34090 | 220/200 | 38602 | 219 | Bosch et al. 2015 |
|  |  | Northern Ahmarian | GrA-54846 | shell | AMS | 39850 | 340/310 | 43527 | 335 | Bosch et al. 2015 |
|  |  | Northern Ahmarian | GrA-57602 | shell | AMS | 36730 | 240/220 | 41335 | 241 | Bosch et al. 2015 |
|  |  | Northern Ahmarian | GrA-57603 | shell | AMS | 38260 | 260/240 | 42390 | 187 | Bosch et al. 2015 |
| 45 | Ksar Akil XVIII | Northern Ahmarian | GrA-57542 | shell | AMS | 36290 | 240/220 | 40921 | 294 | Bosch et al. 2015 |
| 45 | Ksar Akil XIX | Northern Ahmarian | GrA-53004 | shell | AMS | 39390 | 330/290 | 43140 | 268 | Bosch et al. 2015 |
| 45 | Ksar Akil XX | Northern Ahmarian | GrA-57597 | shell | AMS | 40040 | 340/300 | 43678 | 348 | Bosch et al. 2015 |
| 43 | Manot Area C Unit 6 | Northern Ahmarian? | RTD-7785.1 | charcoal | ABA | 32410 | 260 | 36331 | 298 | Alex et al. 2016 |
|  |  | Northern Ahmarian? | RTD-7785.2 | charcoal | ABA | 32900 | 150 | 36947 | 333 | Alex et al. 2016 |
|  |  | Northern Ahmarian? | RTD-7786.1 | charcoal | ABA | 28940 | 180 | 33136 | 258 | Alex et al. 2016 |
|  |  | Northern Ahmarian? | RTD-7786.2 | charcoal | ABA | 28850 | 100 | 33059 | 200 | Alex et al. 2016 |
|  |  | Northern Ahmarian? | RTD-7086 | charcoal | WBA | 38880 | 310 | 42777 | 220 | Alex et al. 2016 |
|  |  | Northern Ahmarian? | RTD-7087 | charcoal | WBA | 41790 | 380 | 45166 | 342 | Alex et al. 2016 |
|  |  | Northern Ahmarian? | RTD-7128B | charcoal | AMS | 28560 | 150 | 32543 | 319 | Alex et al. 2016 |
|  |  | Northern Ahmarian? | RTD-7118 | charcoal | ABA | 40280 | 320 | 43863 | 344 | Alex et al. 2016 |
|  |  | Northern Ahmarian? | RTD-7119 | charcoal | ABA | 42310 | 380 | 45594 | 345 | Alex et al. 2016 |
|  |  | Northern Ahmarian? | RTD-7130B | charcoal | AMS | 30860 | 180 | 34779 | 171 | Alex et al. 2016 |
|  |  | Northern Ahmarian? | RTD-7117 | charcoal | ABA | 41610 | 540 | 45009 | 468 | Alex et al. 2016 |
|  |  | Northern Ahmarian? | RTD-7129B | charcoal | AMS | 31270 | 190 | 35162 | 224 | Alex et al. 2016 |
|  |  | Northern Ahmarian? | RTD7197.1 | charcoal | ABA | 37330 | 300 | 41786 | 228 | Alex et al. 2016 |
|  |  | Northern Ahmarian? | RTD7197.2 | charcoal | ABA | 37120 | 300 | 41638 | 243 | Alex et al. 2016 |
| 43 | Manot Area C Unit 7 | Northern Ahmarian? | RTD-7115 | charcoal | ABA | 42210 | 390 | 45511 | 350 | Alex et al. 2016 |
|  |  | Northern Ahmarian? | RTD-7127B | charcoal | AMS | 25080 | 110 | 29122 | 172 | Alex et al. 2016 |
|  |  | Northern Ahmarian? | RTD-7196 | charcoal | ABA | 41100 | 450 | 44575 | 423 | Alex et al. 2016 |
| 9 | Al-Ansab 1 | Southern Ahmarian | AN001 | charcoal | ABA-AMS | 32869 | 409 | 37071 | 617 | this paper |
|  |  | Southern Ahmarian | AN002 | charcoal | ABA-AMS | 33041 | 419 | 37257 | 635 | this paper |
|  |  | Southern Ahmarian | AN003 | charcoal | ABA-AMS | 33292 | 432 | 37499 | 640 | this paper |
|  |  | Southern Ahmarian | AN004 | charcoal | ABA-AMS | 33564 | 444 | 37756 | 640 | this paper |
|  |  | Southern Ahmarian | AN005 | charcoal | ABA-AMS | 33552 | 460 | 37740 | 655 | this paper |
|  |  | Southern Ahmarian | AN006 | charcoal | ABA-AMS | 32927 | 439 | 37141 | 649 | this paper |
|  |  | Southern Ahmarian | AN007 | charcoal | ABA-AMS | 33447 | 440 | 37643 | 640 | this paper |
| 17 | Qseimeh I | Southern Ahmarian | DRI-2965 | ostrich | ABA | 34010 | 510 | 38322 | 689 | Gilead 1984 |
| 15 | Qadesh Barnea 501 | Southern Ahmarian | Pta-2819 | ostrich | ABA | 33800 | 940 | 38030 | 1160 | Goring-Morris and Belfer-Cohen 2003 |
| 14 | Qadesh Barnea 601B | Southern Ahmarian | Pta-2964 | ostrich | ABA | 32470 | 780 | 36666 | 993 | Goring-Morris and Belfer-Cohen 2003 |
|  | A296 | Southern Ahmarian | Pta-2941 | ostrich | ABA | 34300 | 1000 | 38578 | 1242 | Bar-Yosef 1985 |
| 49 | Umm el-Tlel 2/level V | Southern Ahmarian? | Gif-900/34 | charcoal | ? | 30310 | 670 | 34356 | 574 | Goring-Morris and Belfer-Cohen 2003 |
| 16 | Qseimeh II | Southern Ahmarian | ? | ? | ? | 30500 | 330 | 34467 | 283 | Phillips 1994, Saca 2002 |
| 2 | Abu Noshra II | Southern Ahmarian | SMU-2122 | charcoal | ABA | 38924 | 1529 | 42948 | 1206 | Phillips and Gladfelter 1989 |
|  |  | Southern Ahmarian | ETH-3076 | charcoal | ABA-AMS | 33940 | 790 | 38177 | 1027 | Phillips 1988 |
|  |  | Southern Ahmarian | ETH-3075 | charcoal | ABA-AMS | 33470 | 680 | 37650 | 853 | Phillips 1994 |
|  |  | Southern Ahmarian | SMU-1762 | charcoal | ABA | 31585 | 2275 | 35862 | 2427 | Phillips 1988 |
|  |  | Southern Ahmarian | SMU-1772 | charcoal | ABA | 31023 | 8537 | 34455 | 8535 | Phillips 1988 |
| 3 | Abu Noshra VI | Southern Ahmarian | SMU-2371 | charcoal | ABA | 31100 | 300 | 35035 | 301 | Phillips 1994 |
| 1 | Abu Noshra I | Southern Ahmarian | SMU-2254 | charcoal | ABA | 35824 | 1090 | 40234 | 1075 | Phillips 1994 |
|  |  | Southern Ahmarian | SMU-2007 | charcoal | ABA | 35805 | 1520 | 40020 | 1481 | Phillips 1994 |
|  |  | Southern Ahmarian | SMU-1824 | charcoal | ABA | 31330 | 2880 | 35569 | 3018 | Phillips 1994 |
|  |  | Southern Ahmarian | B-13898 | charcoal | ABA | 29580 | 1610 | 33552 | 1620 | Phillips 1988 |
|  |  | Southern Ahmarian | B-13897 | sediment | ? | 25950 | 360 | 30128 | 432 | Phillips 1994 |
| 28 | Boker A | Southern Ahmarian | SMU-578 | charcoal | ABA | 37920 | 2810 | 41696 | 2603 | Weinstein 1984 |
| 22 | Lagama VII | Southern Ahmarian | SMU-172 | charcoal | ABA | 34170 | 3670 | 38075 | 3492 | Haas and Haynes 1975; Haas 1977 |
|  |  | Southern Ahmarian | SMU-185 | charcoal | ABA | 31210 | 2780 | 35456 | 2934 | Haas and Haynes 1975; Haas 1977 |
| 24 | Lagama VIII | Southern Ahmarian | SMU-119 | ostrich | ? | 32980 | 2410 | 37239 | 2482 | Housley 1994 |

Alex, B., Barzilai, O., Hershkovitz, I., Marder, O., Berna, F., Caracuta, V., Abulafia, T., Davis, L., Goder-Goldberger, M., Lavi, R., Mintz, E., Regev, L., Bar-Yosef Mayer, D., Tejero, J.-M., Yeshurun, R., Ayalon, A., Bar-Matthews, M., Yasur, G., Frumkin, A., Latimer, B., Hans, M.G., Boaretto, E., Radiocarbon chronology of Manot Cave, Israel and Upper Paleolithic dispersals. Science Advances; 2016, 3: e1701450.

Bar-Yosef, O., The Stone Age of the Sinai Peninsula. In: M. Leverani, A. Palimeri, R. Peroni (eds.), Studi di paletnologia in orone di Salvatore Puglisi. Roma: Universita Di Roma "La Sapienza"; 1985. pp. 107-122.

Bar-Yosef, O., Arnold, M., Belfer-Cohen, A., Goldberg, P., Housley, R., Laville, H., Meignen, L., Mercier, N., Vogel, J.C., Vandermeersch, B., The dating of the Upper Palaeolithic layers in Kebara Cave, Mt. Carmel. J. Arch. Sci. 1996; 23: 297–306.

Bar-Yosef, O., Belfer-Cohen, A., The Qafzeh Upper Paleolithic assemblages: 70 years later. Eurasian Prehistory 2004; 2: 145–180.

Bosch, M.D., Mannino, M.A., Prendergast, A.L., O’Connell, T.C., Demarchi, B., Taylor, S.M. et al., New chronology for Ksâr ’Akil (Lebanon) supports Levantine route of Modern Human dispersal into Europe. PNAS 2015; 112(25): 7683–7688.

Douka, K., Exploring “the great wilderness of prehistory”: The Chronology of the Middle to the Upper Paleolithic Transition in the Northern Levant. Mitteilungen der Gesellschaft für Urgeschichte 2013; 22: 11-40.

Douka, K., Bergman, C.A., Hedges, R.E.M., Wesselingh, F.P., Higham, T.F.G., Chronology of Ksar Akil (Lebanon) and implications for the colonization of Europe by Anatomically Modern Humans. PLoS ONE 2013; 8(9), doi: 10.1371/journal.pone.0072931

Gilead, I., Paleolithic sites in Northeastern Sinai. Paléorient 1984; 10: 135-142.

Goring-Morris, A.N., Belfer-Cohen, A. (eds.), More than Meets the Eye. Studies on Upper Palaeolithic diversity in the Near East. Oxford: Oxbow Books; 2003.

Haas, H.. Radiocarbon dating of charcoal and ostrich egg shells from Mushabi and Lagama sites. In: O. Bar-Yosef, J.L. Phillips (eds.), Prehistoric Investigations in Gebel Maghara, Northern Sinai. Jerusalem: Quedem 1977. pp. 261-264.

Haas, H., Haynes, V., Southern Methodist University radiocarbon date list II. Radiocarbon 1975; 17(3): 354-363.

Housley, R.A., Eastern Mediterranean chronologies. The Oxford AMS contribution. In: O. Bar-Yosef, R.S. Kra (eds.), Late Quaternary chronology and paleoclimates of the Eastern Mediterranean. Radiocarbon 1994; 36: 55-73.

Hedges, R.E.M., Housley, R.A., Law, I. A., Bronk, R.C., Radiocarbon dates from the Oxford AMS system: Archaeometry Datelist 10. Archaeometry 1992; 32: 101-108.

Lengyel, G., Boaretto, E., Fabre, L., Ronen, A, New AMS ^14^C dates from the Early Upper Paleolithic sequence of Raqefet Cave, Mount Carmel, Israel. Radiocarbon 2006; 42(2): 253-258.

Mellars, P., Tixier J., Radiocarbon-accelerator dating of Ksar Akil (Lebanon) and the chronology of the Upper Paleolithic sequence in the Middle East. Antiquity 1989; 63: 761-768.

Phillips, J., The Upper Paleolithic of the Wadi Feiran, Southern Sinai. Paléorient 1988; 14: 183-200.

Phillips, J.L., The Upper Paleolithic chronology of the Levant and the Nile Valley. In: O. Bar-Yosef, R.S. Kra (eds.), Late Quaternary chronology and paleoclimates of the Eastern Mediterranean. Radiocarbon 1994; 36: 169-176.

Phillips, J.L., Gladfelter, B.G., A survey in the Upper Wadi Feiran Basin, Southern Sinai. Paléorient 1989; 15(2): 113-122.

Rebollo, N. R., Weiner, S., Brock, F., Meignen, L., Goldberg, P., Belfer-Cohen, A., Bar-Yosef, O., Boaretto, E., New radiocarbon dating of the transition from the Middle to the Upper Paleolithic in Kebara Cave, Israel. Journal of Archaeological Science 2011; 38: 2424–2433.

Saca, I.N., Reconfiguring the early Upper Paleolithic of the southern Levant: The bigger picture unpubl. PhD Thesis, University of Illinois at Chicago (Chicago 2002).

Stutz, A.J., Shea, J.J., Rech, J.A., Pigati, J.S., Wilson, J., Belmaker, M., Albert, R.M., Arpin, T., Cabanes, D., Clark, J.L., Hartman, G., Hourani, F., White, C.E., Nilsson Stutz, L., Early Upper Paleolithic chronology in the Levant: new ABOx-SC accelerator mass spectrometry results from the Mughr el-Hamamah Site, Jordan. Journal of Human Evolution 2015; 85: 157-173.

Weinstein, J.M., Radiocarbon dating in the southern Levant. Radiocarbon 1984; 26(3): 297-366.
