# Supplementary material for: Al-Ansab and the Dead Sea: Mid-MIS 3 archaeology and environment of the early Ahmarian population of the Levantine corridor
Source: PLoS One. 2020 Oct 13;15(10):e0239968. doi: 10.1371/journal.pone.0239968 (PMC7553344; doi:10.1371/journal.pone.0239968)
Supplement: S4 Table — This table contains data from the material excavated in the campaigns 2009 until 2014. (DOCX) [file pone.0239968.s004.docx]

| Blank counts | URC Ansab |  | URC General | | AHP |  | Silicified Limestone | | Unknown source | | Total |  |
| --- | --- | --- | --- | --- | --- | --- | --- | --- | --- | --- | --- | --- |
|  | n | % | n | % | n | % | n | % | n | % | n | % |
| Flakes | 427 | 32.3 | 14 | 32.6 | 66 | 20.4 | 107 | 29.6 | 1 | 20 | 615 | 6.3 |
| Blades | 394 | 29.8 | 26 | 60.5 | 65 | 20.1 | 72 | 19.9 | 4 | 80 | 561 | 5.7 |
| Bladelets | 443 | 33.5 | 1 | 2.3 | 178 | 55.1 | 176 | 48.8 | 0 | 0 | 798 | 8.2 |
| Blade (-lets) | 843 | 63.8 | 27 | 62.8 | 243 | 75.2 | 252 | 69.8 | 4 | 80 | 1369 | 14 |
| cores | 26 | 2 | 2 | 4.7 | 9 | 2.8 | 1 | 0.3 | 0 | 0 | 38 | 0.4 |
| cores on flakes | 6 | 0.5 | 0 | 0 | 0 | 0 | 0 | 0 | 0 | 0 | 6 | 0.1 |
| carinated pieces | 4 | 0.3 | 0 | 0 | 0 | 0 | 1 | 0.3 | 0 | 0 | 5 | 0.1 |
| debitage | 1306 | 98.8 | 43 | 100 | 318 | 98.5 | 361 | 100 | 5 | 100 | 2033 | 20.8 |
| chunks (not classified by raw material) | | |  |  |  |  |  |  |  |  | 339 | 3.5 |
| chips (not classified by raw material) | | |  |  |  |  |  |  |  |  | 6056 | 61.9 |
|  | | |  |  |  |  |  |  |  |  |  |  |
| debris |  |  |  |  |  |  |  |  |  |  | 6395 | 65.3 |
| total | 1322 | 100 | 43 | 100 | 323 | 100 | 361 | 100 | 5 | 100 | 9787 | 100 |
|  |  |  |  |  |  |  |  |  |  |  |  |  |
| tools | 47 | 3.6 | 3 | 7 | 8 | 2.5 | 4 | 1.1 | 0 | 0 | 62 | 0.6 |
| CTEs | 97 | 7.3 | 6 | 14 | 8 | 2.5 | 10 | 2.8 | 0 | 0 | 121 | 1.2 |
