# Supplementary material for: Al-Ansab and the Dead Sea: Mid-MIS 3 archaeology and environment of the early Ahmarian population of the Levantine corridor
Source: PLoS One. 2020 Oct 13;15(10):e0239968. doi: 10.1371/journal.pone.0239968 (PMC7553344; doi:10.1371/journal.pone.0239968)
Supplement: S5 Table — (DOCX) [file pone.0239968.s005.docx]

| **Name** | **Provider** | **Type** | **Access date** | **Licence** |
| --- | --- | --- | --- | --- |
| ASTER V3 | USGS | Raster | 2019 | No redistribution requirements;  https://lpdaac.usgs.gov/data/data-citation-and-policies/ |
| Land Polygons | Naturalearthdata | Polygon | 2019 | Public domain  https://www.naturalearthdata.com/about/ |
| MIS 3 sea-level | CRC 806 | Polygon | 2019 | CRC 806 own data (CC BY 4.0)  https://crc806db.uni-koeln.de/dataset/show/paleocoastlines-gis-dataset1462293239/ |
