# Supplementary material for: Al-Ansab and the Dead Sea: Mid-MIS 3 archaeology and environment of the early Ahmarian population of the Levantine corridor
Source: PLoS One. 2020 Oct 13;15(10):e0239968. doi: 10.1371/journal.pone.0239968 (PMC7553344; doi:10.1371/journal.pone.0239968)
Supplement: S6 Table — (DOCX) [file pone.0239968.s006.docx]

| **Topic** | **Author** | **URL** |
| --- | --- | --- |
| Sedimentological and geochemical analsysis of the Wadi Sabra | Bertrams et al. | https://crc806db.uni-koeln.de/dataset/show/b1jordansedimentology-and-geochemistry-bertrams-et-al-2012qi/ |
| Luminescence dating project Jordan | Klasen et al. | https://crc806db.uni-koeln.de/dataset/show/luminescence-dating-project-b1-jordan/ |
